# Exosome-derived circCCAR1 promotes CD8 + T-cell dysfunction and anti-PD1 resistance in hepatocellular carcinoma

**DOI:** 10.1186/s12943-023-01759-1

**Published:** 2023-03-18

**Authors:** Zongqiang Hu, Gang Chen, Yingpeng Zhao, Hongqiang Gao, Laibang Li, Yanfeng Yin, Jie Jiang, Li Wang, Yuanyi Mang, Yang Gao, Shengning Zhang, Jianghua Ran, Li Li

**Affiliations:** Department of Hepato-Pancreato-Biliary Surgery, First People’s Hospital of Kunming City & Calmette Affiliated Hospital of Kunming Medical University, 1228 Beijing Road, Panlong District, Kunming, 650032 Yunnan China

**Keywords:** circRNA, CCAR1, Hepatocellular carcinoma, Exosome, Anti-PD1

## Abstract

**Background:**

Circular RNAs (circRNAs) can be encapsulated into exosomes to participate in intercellular communication, affecting the malignant progression of a variety of tumors. Dysfunction of CD8 + T cells is the main factor in immune escape from hepatocellular carcinoma (HCC). Nevertheless, the effect of exosome-derived circRNAs on CD8 + T-cell dysfunction needs further exploration.

**Methods:**

The effect of circCCAR1 on the tumorigenesis and metastasis of HCC was assessed by in vitro and in vivo functional experiments. The function of circCCAR1 in CD8 + T-cell dysfunction was measured by enzyme-linked immunosorbent assay (ELISA), western blotting and flow cytometry. Chromatin immunoprecipitation, biotinylated RNA pull-down, RNA immunoprecipitation, and MS2 pull-down assays were used to the exploration of mechanism. A mouse model with reconstituted human immune system components (huNSG mice) was constructed to explore the role of exosomal circCCAR1 in the resistance to anti-PD1 therapy in HCC.

**Results:**

Increased circCCAR1 levels existed in tumor tissues and exosomes in the plasma of HCC patients, in the culture supernatant and HCC cells. CircCCAR1 accelerated the growth and metastasis of HCC in vitro and in vivo. E1A binding protein p300 (EP300) and eukaryotic translation initiation factor 4A3 (EIF4A3) promoted the biogenesis of circCCAR1, and Wilms tumor 1-associated protein (WTAP)-mediated m6A modification enhanced circCCAR1 stability by binding insulin-like growth factor 2 mRNA-binding protein 3 (IGF2BP3). CircCCAR1 acted as a sponge for miR-127-5p to upregulate its target WTAP and a feedback loop comprising circCCAR1/miR-127-5p/WTAP axis was formed. CircCCAR1 is secreted by HCC cells in a heterogeneous nuclear ribonucleoprotein A2/B1 (hnRNPA2B1)-dependent manner. Exosomal circCCAR1 was taken in by CD8 + T cells and caused dysfunction of CD8 + T cells by stabilizing the PD-1 protein. CircCCAR1 promoted resistance to anti-PD1 immunotherapy. Furthermore, increased cell division cycle and apoptosis regulator 1 (CCAR1) induced by EP300 promoted the binding of CCAR1 and β-catenin protein, which further enhanced the transcription of PD-L1.

**Conclusions:**

The circCCAR1/miR-127-5p/WTAP feedback loop enhances the growth and metastasis of HCC. Exosomal circCCAR1 released by HCC cells contributes to immunosuppression by facilitating CD8 + T-cell dysfunction in HCC. CircCCAR1 induces resistance to anti-PD1 immunotherapy, providing a potential therapeutic strategy for HCC patients.

**Supplementary Information:**

The online version contains supplementary material available at 10.1186/s12943-023-01759-1.

## Introduction

Hepatocellular carcinoma (HCC) has become the fifth most malignant tumor worldwide, seriously affecting patient quality of life [1]. HCC is a highly fatal tumor that is usually found in the advanced stage [2]. In recent years, immune checkpoint blocking (ICB) therapy, specifically antibodies against programmed cell death 1 (PD1)/ programmed cell death-Ligand 1 (PD-L1) signaling, has been widely used in cancer, which greatly prolongs the survival time of cancer patients [3]. A PD1 antibody has been approved for second-line therapy in advanced HCC [4]. However, only 17–18% of advanced HCC patients have achieved complete or partial response to anti-PD1 antibody therapy [5]. Therefore, there is an urgent need to explore the mechanism of tolerance to anti-PD1 therapy and to identify a combination therapy strategy to improve its effectiveness in HCC treatment.

Impaired antitumor immunity featured by existing dysfunctional CD8 + T cells in the tumor microenvironment (TME) is a characteristic of cancer [6]. Long-term persistence of tumor antigens and/or the suppressive TME actuate antitumor effector CD8 + T cells to a dysfunctional state called ‘T cell exhaustion’ [7]. Exhausted CD8 + T cells highly express inhibitory receptors, such as PD1, T-cell immunoglobulin and mucin-domain containing-3 (TIM3), lymphocyte-activation gene 3 (LAG3) and T cell immunoreceptor with Ig and ITIM domains (TIGIT). Moreover, exhausted CD8 + T cells appear decreased proliferative capacity, subdued cytotoxic function, and emasculated ability to release effector cytokines [8]. There are 2 major varieties of exhausted CD8 + T cells in TME: fully exhausted cells expressing high levels of PD1 on the cell surface, and partially exhausted cells with intermediate levels of PD1 on the cell surface [7]. The function of partially exhausted CD8 + T cells could be partially rescued by immune checkpoint blockers, such as anti-PD1 or anti-PDL1 antibodies, which is currently one of the treatment strategies for cancer [7]. Recent studies suggest that several key genes participate in CD8 + T cell exhaustion, such as thymocyte selection associated high mobility group box (TOX) [9, 10], MYB [11], P-selectin glycoprotein ligand-1 (PSGL-1) [12] and regulator of G protein signaling 16 (Rgs-16) [13]. However, whether other factors are involved in regulating CD8 + T cell dysfunction in the TME remains more exploration.

Circular RNAs (circRNAs), characterized as noncoding RNAs with a closed‐loop structure, regulate biological processes by acting as sponges for microRNAs (miRNAs) or binding to proteins [14, 15]. Many circRNAs are involved in cell proliferation, differentiation, apoptosis, invasion, stemness and resistance to therapy of HCC [16–18]. For instance, Huang et al. indicated that circular RNA circMET drives immunosuppression and anti-PD1 therapy resistance in HCC via the miR-30-5p/snail/dipeptidyl peptidase 4(DPP4) axis [19]. CircMRPS35 promotes cisplatin resistance [20] and circRNA-SORE sustains sorafenib resistance in HCC [21]. CircRNAs also were reported to be packaged into exosomes and related to the malignant process and immunosuppression of HCC. Exosomal circUHRF1 released by HCC cells can promote natural killer cell dysfunction, leading to resistance to anti-PD1 therapy in HCC patients [22]. Exosomal circGSE1 facilitates the immune evasion of HCC through promoting the expansion of regulatory T cells [23]. Exosomal circTMEM181 avails the immunosuppressive microenvironment and endows anti-PD1 resistance in HCC by upregulating CD39 expression in macrophages [24]. Nevertheless, the underlying functional and regulatory mechanisms of HCC‐derived exosomal circRNAs in immunosuppressive microenvironment, especially its role on CD8 + T cells, still needs more exploration.

N6-methyladenosine (m6A) modification, one of the major posttranscriptional modifications of eukaryotic RNAs [25]. Dysregulation of m6A profiles have been implicated in the carcinogenesis and progression of HCC [26–28]. In recent years, a growing body of research shows that m6A-modified circRNAs paly important role in HCC. For instance, m6A-mediated upregulation of circMDK promotes tumorigenesis in HCC [29]. m6A-modified circRNA-SORE sustains sorafenib resistance in HCC [21]. Fat mass and obesity-associated protein (FTO) mediated m6A demethylation of circGPR137B inhibits tumorigenesis and metastasis of HCC [30]. Nevertheless, the roles of m6A-modified circRNAs in regulating the antitumor immunity of HCC still need further investigations.

In this study, we first identified a novel circ_0000240 (circCCAR1) and found that circCCAR1/miR-127-5p/WTAP feedback loop enhances the growth and metastasis of HCC. Exosomal circCCAR1 released by HCC cells enhanced HCC resistance to anti-PD1 therapy by facilitating CD8 + T-cell dysfunction by preventing PD1 degradation. Thus, our results revealed the function of circCCAR1 in the progression of HCC and lay a foundation for studying the function of circRNAs in the immunotherapy of HCC.

## Materials and methods

### Clinical samples

From the First People's Hospital of Kunming City, tumor tissues, adjacent normal tissues, and blood samples were collected from 58 HCC patients. The clinical characteristics of these HCC patients are shown in Table S1. The study was approved by the Ethics Committee of the First People's Hospital of Kunming City. Written informed consent was obtained from all patients.

### Quantitative real-time polymerase chain reaction (qRT‒PCR) and genomic DNA extraction

TRIzol reagent (Beyotime, Shanghai, China) was used to extract total RNA. BeyoFast™ SYBR Green One-Step qRT‒PCR Kit (Beyotime) was used to detect targeting gene according to the manual. In addition, for miRNAs, MicroRNA Reverse Transcription Kit (Takara Biotechnology, Japan) were used to perform reverse transcription. qRT-PCR was carried out on ABI 7500 fast PCR System (Carlsbad, CA, USA) with a SYBR green PCR Master Mix (TOYOBO, Japan). β-actin and U6 applied as internal references for mRNAs and miRNAs. The relative expressions were calculated with 2^–ΔΔCT^ method. Moreover, genomic DNA (gDNA) was extracted by a Universal Genomic DNA Purification Mini Spin Kit (Beyotime) based on the manufacturer’s protocol. All primers were synthesized by GenePharma (Shanghai, China) and are listed in Table S2.

### Chromatin immunoprecipitation (ChIP) assay

After crosslinking with 1% (v/v) formaldehyde (Sigma‒Aldrich) and stopping with 125 mM glycine, the cells were resuspended and sonicated. Chromatin extracts were immunoprecipitated with protein A/G-plus agarose beads (YEASEN, Shanghai, China) pre-coated with corresponding antibodies or IgG. After washing, elution and decrosslinking, the immunoprecipitated DNA was detected by PCR.

### RNA immunoprecipitation (RIP) assay

RIP assay was performed by using a Magna RIP RNA-Binding Protein Immunoprecipitation Kit (Millipore). Briefly, HCC cells were harvested and lysed in RIP lysis buffer on ice for 30 min. After centrifugation, the supernatant was incubated with 30 μL of Protein-A/G agarose beads (Roche, USA) and antibodies (anti-Ago2, EIF4A3, WTAP, IGF2BP1, IGF2BP2, IGF2BP3, hnRNPA2B1 or PD1). IgG was used as a negative control. After overnight incubation, the immune complexes were centrifuged then washed six times with washing buffer. The beads-bound proteins were further analyzed using Western blotting. The immunoprecipitated RNA was applied to qRT-PCR analysis.

### Biotinylated RNA pull-down assay

The biotin-labeled circCCAR1 and control probes were synthesized by GenePharma. Formaldehyde (1%) and glycine buffer were used to cross-link and balance cells. Total protein was extracted by Lysis buffer. The probes mixed with streptavidin agarose beads (Sigma‒Aldrich) in RIP buffer for 4 h. Then, the HCC cell lysates were incubated with the mixture overnight at 4 °C. After purification, the bound proteins were identified by Western blotting.

### MS2 pull-down assay

The recombinant plasmids pCCAR1-MS2, pEmpty-MS2, and pMS2-GST were constructed by GenePharma. HCCLM3 cells were plated overnight and then cotransfected with pCCAR1-MS2 vector or pEmpty-MS2 and pMS2-GST for 48 h of incubation. After collection and quantification of proteins in cells, 500 μl (2 μg/μl) lysate was incubated with GSH agarose beads (Thermo Fisher Scientific) for 4 h at 4 °C. After washing, bound proteins were isolated and detected by Western blotting.

### Methylated RNA immunoprecipitation (MeRIP)

The GenSeq™ m6A RNA IP Kit (GenSeq Inc., China) was used to examine m6A modifications in individual genes according to the manual. After fragmentation and purification, 200 μg of total RNA was incubated with m6A antibody- or mouse IgG-conjugated Protein A/G Magnetic Beads in 500 μl IP buffer overnight. Methylated RNAs were immunoprecipitated, eluted and measured by qRT‒PCR.

### CD8 + T-cell isolation

Human CD8 + T cells were purified from healthy donor peripheral blood mononuclear cells by a CD8 + Cell Positive Selection Kit (ImunoSep, Precision BioMedicals Co., Ltd., China) according to the manual. For CD8 + T-cell activation, cells were incubated with CD3/CD28 Dynabeads (2 µl/well) and IL2 (20 ng/mL) for 48 h.

### In vivo tumor growth assay

All animal experiments were approved by the Animal Care Committee of Kunming Medical University. BALB/c nude male mice (5–6 weeks old) and Humanized NOD/LtSz-scid/IL2Rγ^null^ (HuNSG) male mice (5–6 weeks old) were obtained to Shanghai Model Organisms Center, Inc. (Shanghai, China). For xenograft studies, HCCLM3 cells (5 × 10^6^ cells per mouse) with circCCAR1 overexpression or knockdown were injected subcutaneously into the right flanks of mice (*n* = 5 for each group). The tumor size was measured every week. After 4 weeks, the mice were sacrificed, and the xenografted tumors were weighed and collected for Ki67 IHC analysis. For pulmonary metastasis studies, HCCLM3 cells (1 × 10^7^ cells per mouse) with circCCAR1 overexpression or knockdown were injected through the lateral tail vein of mice (*n* = 9 for each group). After 6 weeks, the mice were sacrificed. Lung tissues were harvested, and metastases were counted. For anti-PD1 therapy experiments, xenograft experiments were performed in HuNSG mice. HCCLM3 cells (5 × 10^6^ cells per mouse) with or without circCCAR1 overexpression were injected into the right flank (*n* = 5 for each group). Then, Opdivo or its isotype control (100 μg per mouse) was injected through the tail vein of mice three times a week for 2 weeks. The day the mice received their first dose was defined as Day 1. The tumor size was measured every 3 days. The levels of exosomal circCCAR1 in the plasma from HuNSG mice were detected at Day 30. The mice were harvested when the tumor volume approached 2000 mm^3^. Tumor volume was calculated using the formula: volume = (length x width^2^)/2. The tumor specimens were surgically removed, fixed, embedded in paraffin, and sectioned. The sections were used for haematoxylin and eosin (H&E) and immunohistochemistry (IHC) staining.

### Immunohistochemistry (IHC)

The IHC assay was performed as previous report [31]. Briefly, tumor tissue samples underwent fixation, paraffin embedding, dewaxing, rehydration, and antigen retrieval. The sections were incubated with Ki67 (ab15580, Abcam) or CD8 (ab237709, Abcam) antibody, stained with secondary antibody, visualized with DAB solution, counterstained with hematoxylin and sealed. Finally, the slides were covered with a cover slip, sealed with neutral balsam (Yeasen, Shanghai, China) and visualized under a fluorescence microscope.

### Statistical analysis

All statistical analyses were performed in GraphPad Prism 6 software and expressed as mean values ± the standard deviation (mean ± SD). Statistical significance was determined by one-way ANOVA test or Student’s t-test. Pearson’s correlation coefficient was used for analyzing statistical correlation. Log-rank tests were used to analyze differences. Differences with *P* < 0.05 were considered significant. Additional experimental methods are described in the Supplementary Materials and Methods.

## Results

### CircCCAR1 is increased in HCC tissues

**Fig. 1:**
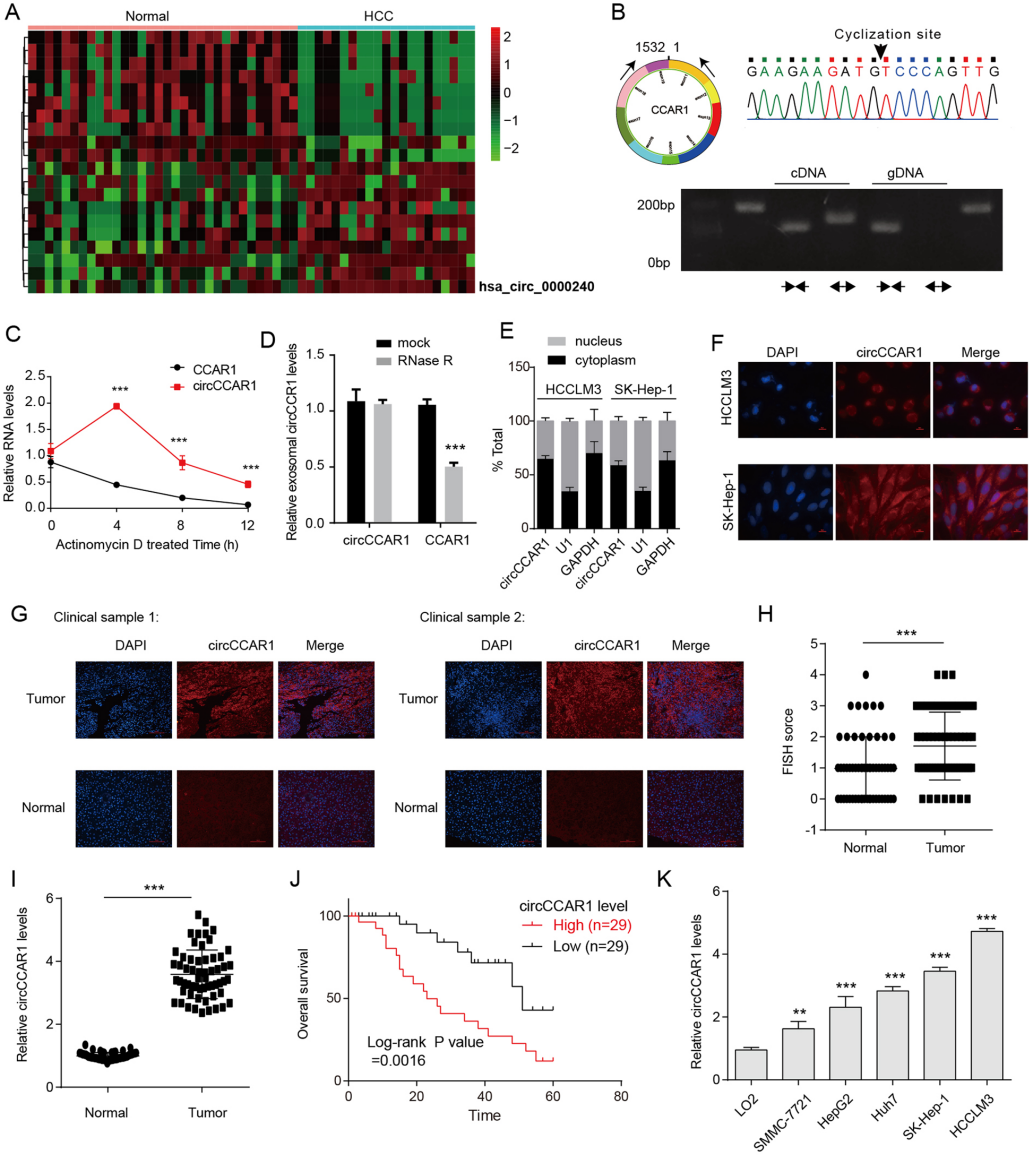
Screening and characterization of circCCAR1 in HCC. **A** The cluster heatmap shows the abnormal circRNAs in serum exosomes of healthy subjects and HCC patients. **B** A PCR assay with divergent primers was used to show the amplification of circCCAR1 from cDNA or genomic DNA (gDNA) of HCCLM3 cells. The back-splice junction sequences were verified by Sanger sequencing. **C** The stability of circCCAR1 and CCAR1 mRNA in HCCLM3 cells after actinomycin D treatment. ***p < 0.001 vs. CCAR1. **D** CCAR1 mRNA and circCCAR1 levels in HCCLM3 cells after RNase R digestion. ***p < 0.001 vs. mock. **E** The intracellular location of circCCAR1 in HCC cells was evaluated by a nuclear–cytoplasmic fractionation assay. **F** The intracellular location of circCCAR1 in HCC cells was evaluated by FISH. **G** Representative fluorescence images of circCCAR1 in HCC tissues and adjacent normal tissues were obtained by FISH. **H** The statistics for FISH. ***p < 0.001. **I** The level of circCCAR1 in HCC tissues and normal tissues was evaluated by qRT‒PCR assay. ***p < 0.001. **J** The association of circCCAR1 expression and overall survival in HCC patients. **K** The level of circCCAR1 in LO2 cells and HCC cell lines was measured. **p < 0.01, ***p < 0.001 vs. LO2

At first, we extracted exosomes from the serum of HCC patients and healthy controls and performed circRNA microarrays to examine their expression profiles. The differentially expressed circRNAs were identified by fold-change filtering (|fold change|> 2) and the Student’s t test (*p* < 0.01), which displayed 117 circRNAs that were significantly differentially expressed in the exosomes from the serum of HCC patients. CircCCAR1 (hsa_circ_0000240) was selected for it is the most elevated circRNA in the exosomes from the serum of HCC patients (Fig. 1A). According to circBase database, circCCAR1 (chr10:70,513,608‐70,532,856) is 1532 bp long and generated from the CCAR1 gene. To determine the existence and circular characteristics of circCCAR1, we designed specific divergent primers for detecting circCCAR1 and specific linear mRNA primers for detecting CCAR1 (Fig. 1B). Then, the specific back-splicing site of circCCAR1 was identified by RT-PCR and Sanger sequencing (Fig. 1B). To examine the stability of circCCAR1, HCCLM3 cells were treated with actinomycin D for indicated time, and circCCAR1 was found to be more stable than linear CCAR1 transcripts (Fig. 1C). Moreover, compared with the linear host genes CCAR1, circCCAR1 was resistant to RNase R digestion (Fig. 1D). Then, the subcellular distribution of circCCAR1 in HCC cell lines was observed by the nuclear/cytoplasmic fractionation assay and RNA fluorescence in situ hybridization (FISH) assay, and found that the majority of circCCAR1 preferentially localized in the cytoplasm (Fig. 1E-F). CircCCAR1 was found to be upregulated in tumor tissues from HCC patients when compared with adjacent normal tissue by FISH and qRT‒PCR assays (Fig. 1G-I). Survival analysis suggested that HCC patients with elevated circCCAR1 level showed an undesirable prognosis (Fig. 1J). The clinical features from 58 cases were collected, and the expression of circCCAR1 was markedly correlated with vascular invasion, tumor grade, TNM stage, and tumor size (Table S1). Moreover, HCC cell lines (SMMC-7721, HepG2, Huh7, SK-Hep-1, and HCCLM3) showed higher expression of circCCAR1 than normal human liver cell line (LO2) (Fig. 1K). To summarize, our data suggest that circCCAR1 is increased in HCC clinical samples and cell lines.

### CircCCAR1 promotes the growth of HCC in vitro and in vivo

**Fig. 2:**
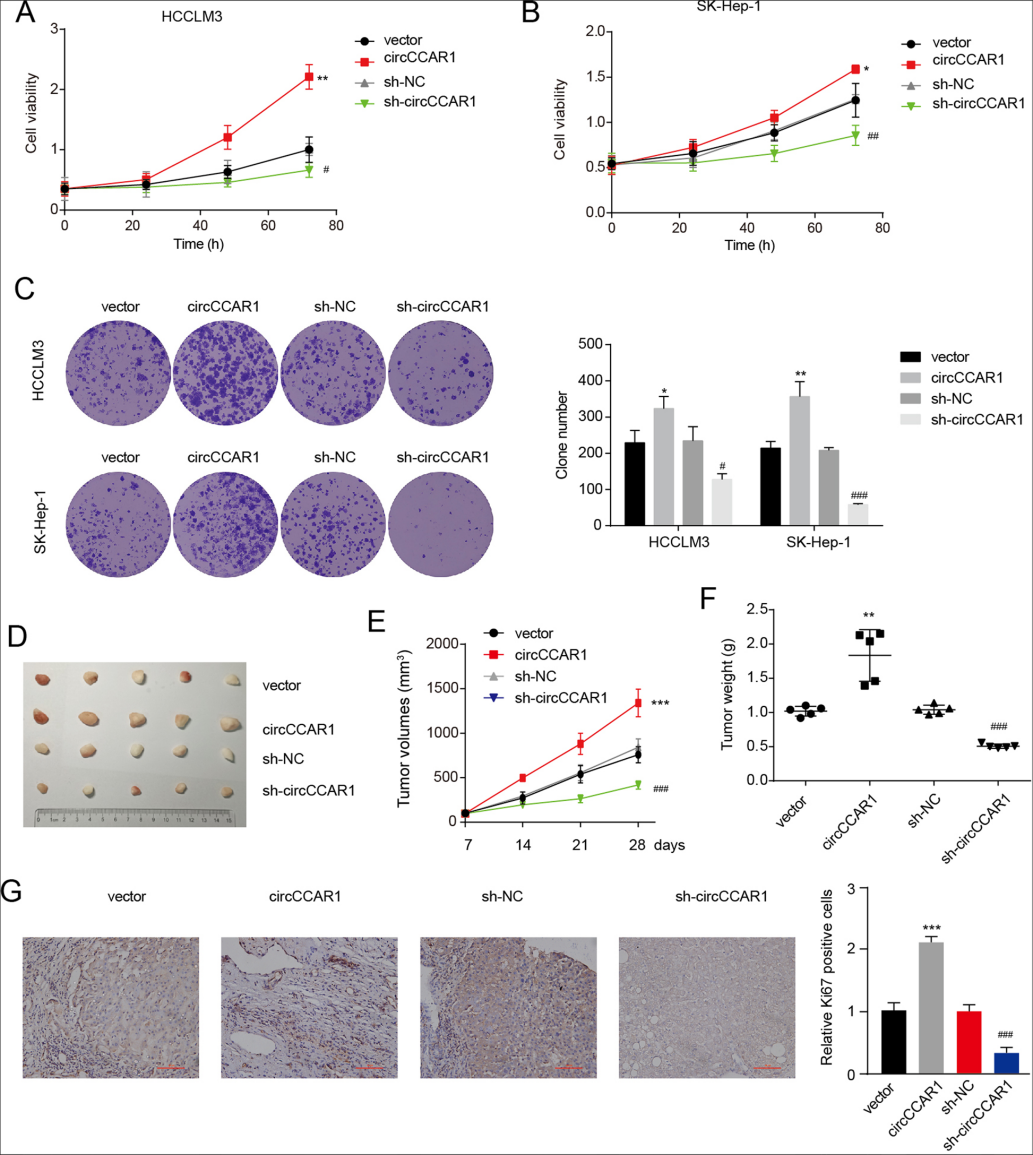
CircCCAR1 promotes HCC growth in vitro and in vivo. **A**-**B** A CCK-8 assay was conducted in HCC cells after circCCAR1 overexpression or depletion. *p < 0.05, **p < 0.01 vs. vector; #*p* < 0.05, ##*p* < 0.01 vs. sh-NC. **C** A colony formation assay was conducted in HCC cells after circCCAR1 overexpression or depletion. *p < 0.05, **p < 0.01 vs. vector; #*p* < 0.05, ###*p* < 0.001 vs. sh-NC. **D** Photograph of xenograft tumors (*n* = 5). **E** Growth curves of xenograft tumors. **F** Tumor weight was determined. **G** Ki67 staining of xenograft tumors. **p < 0.01, ***p < 0.001 vs. vector; ###*p* < 0.001 vs. sh-NC

To study the possible function of circCCAR1 on biological processes of in HCC, circCCAR1 was depleted or increased in HCC cells by shRNA targeting the back-splicing site of circCCAR1 and an overexpression vector of circCCAR1. The shRNA for circCCAR1 significantly decreased and the overexpression vector of circCCAR1 significantly upregulated circCCAR1 levels in HCC cells (Fig. S1A). Importantly, the overexpression and knockdown system did not affect the mRNA and protein expression of CCAR1 (Fig. S1B-C). CCK8 assay revealed that knockdown of circCCAR1 in HCC cells caused significant suppression of cell viability (Fig. 2A-B). Overexpression of circCCAR1 markedly facilitated the proliferation of HCCLM3 and SK-Hep-1 cells. Furthermore, downregulation of circCCAR1 impaired the colony forming ability of HCCLM3 and SK-Hep-1 cells, whereas overexpression of circCCAR1 had the opposite effect (Fig. 2C). Then, we investigated the role of circCCAR1 in HCC cell growth in vivo. Stable HCCLM3 cells with upregulation or downregulation of circCCAR1 were constructed first (Fig. S1D). These stable HCCLM3 cells were used to establish HCC xenograft models. Overexpression of circCCAR1 promoted the growth of xenograft tumors, whereas circCCAR1 depletion inhibited tumor growth in vivo (Fig. 2D-F). The xenografts derived from circCCAR1-overexpressing cells showed increased expression of Ki67, while circCCAR1 knockdown exhibited the opposite effect (Fig. 2G). These data indicate that circCCAR1 facilitates the growth of HCC in vitro and in vivo.

### CircCCAR1 promotes the metastasis of HCC in vitro and in vivo

**Fig. 3:**
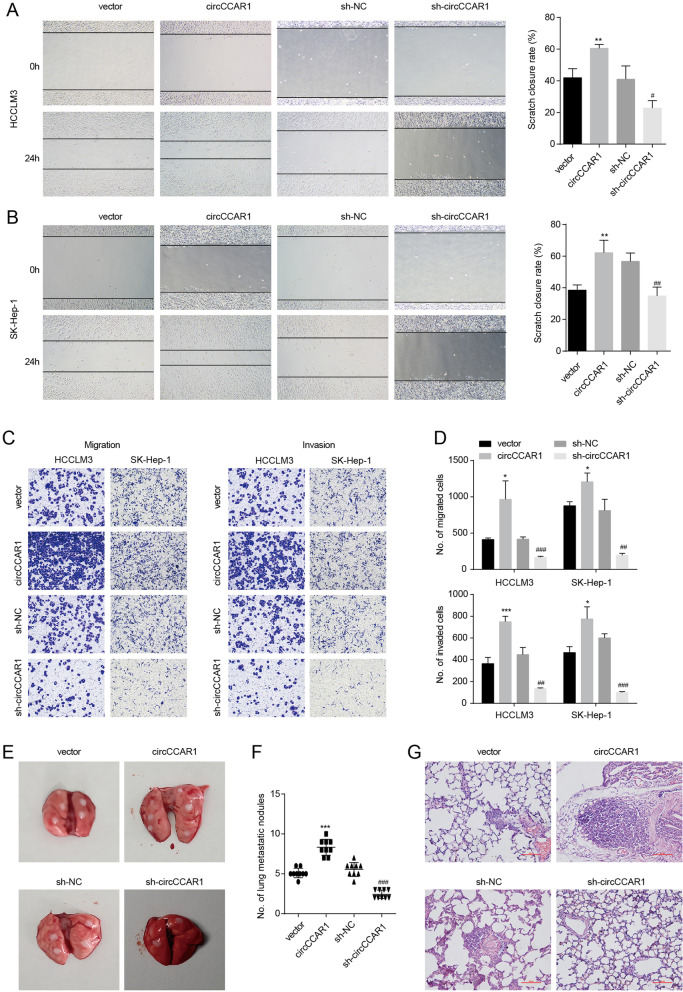
CircCCAR1 promotes HCC metastasis in vitro and in vivo. **A-B** The migration capacity of HCC cells was evaluated by wound‐healing assays. **C-D** The migration and invasion abilities of HCC cells were evaluated using Transwell assays. **E** Gross observation of lung metastases in mice *(n* = 9). **F** The number of metastatic nodules was counted. **G** Lung sections were stained with H&E. *p < 0.05, **p < 0.01, ***p < 0.001 vs. vector; #*p* < 0.05, ##*p* < 0.01, ###*p* < 0.001 vs. sh-NC

Metastasis is a hallmark for cancer and the metastasis of HCC seriously harms the prognosis of HCC patients. We then investigated the effect of circCCAR1 on the migration and invasion of HCC cells. The function of circCCAR1 on the migration of HCC cells was assessed by wound healing and Transwell migration assays. The results displayed that circCCAR1 depletion reduced migration of HCC cells, and circCCAR1 overexpression strengthened migration of HCC cells (Fig. 3A-C). Moreover, Transwell invasion assays suggested that overexpression of circCCAR1 increased and knockdown of circCCAR1 expression decreased the invasion ability of HCC cells (Fig. 3D). To evaluate circCCAR1 in HCC metastasis in vivo, stable HCCLM3 cells with circCCAR1 overexpression or depletion were intravenously injected into the tail vein of BALB/c nude mice. Lung tissues were harvested and the number of metastases was counted. The data indicated that 100% (9/9) of the mice in the circCCAR1-overexpressing group had metastatic nodules, while metastatic nodules were ultimately found in 44% (4/9) of mice in the vector group (Fig. 3E). Metastatic nodules were found in only 11% (1/9) of mice in the circCCAR1 depletion group. In addition, the number of tumor nodules was greater in the circCCAR1-overexpressing group and was smaller in the circCCAR1-depleted group (Fig. 3E-G). In summary, these data revealed the significance of circCCAR1 in accelerating the aggressiveness of HCC cells.

### EP300-mediated H3K27ac promotes circCCAR1 expression in HCC

**Fig. 4:**
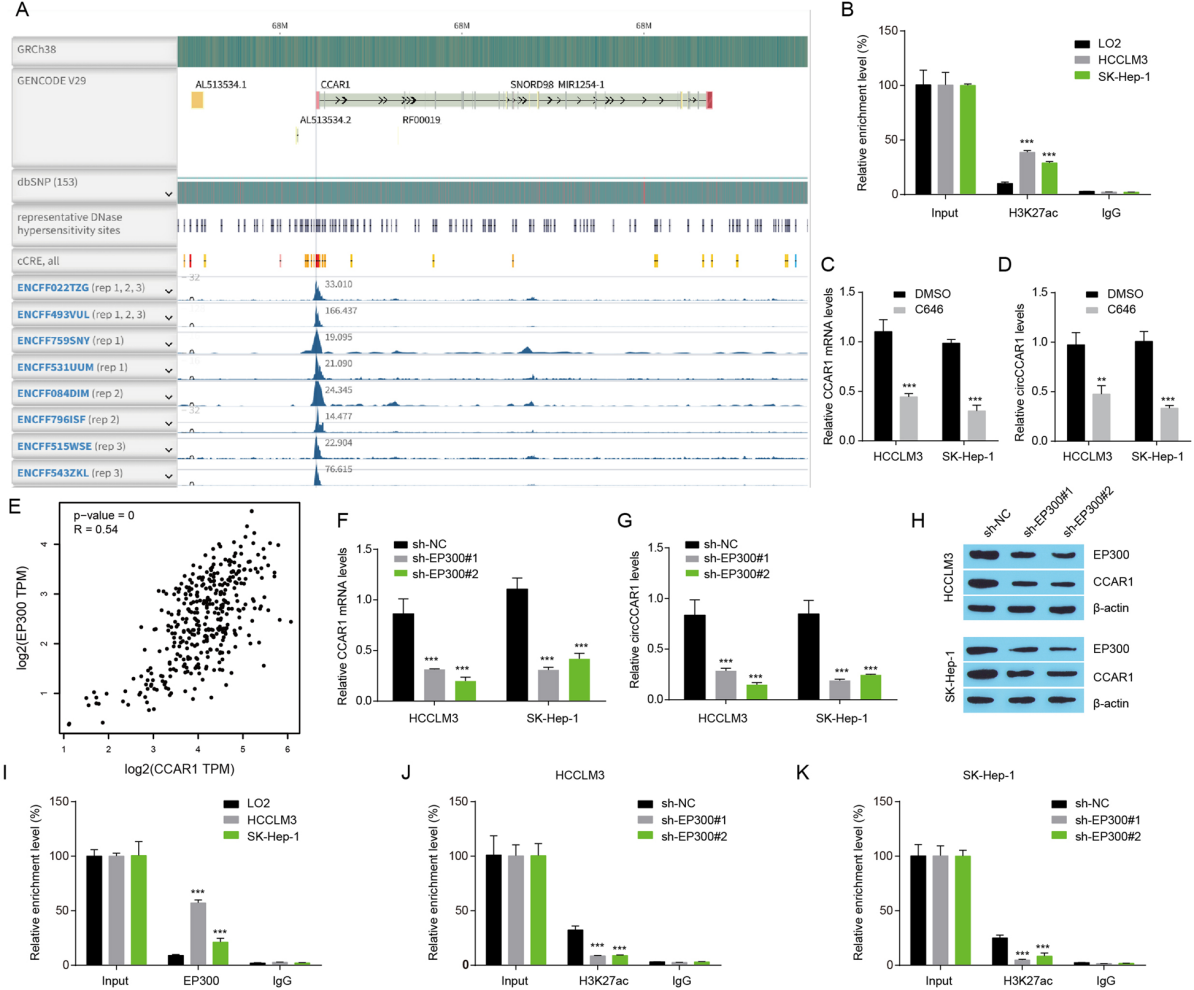
EP300 knockdown inhibits circCCAR1 expression. **A** The H3K27ac signals in the CCAR1 promoter region in HepG2 cells were analyzed using ENCODE. **B** The H3K27ac signals in the CCAR1 promoter were measured using ChIP‒qPCR assay in LO2, HCCLM3 and SK-Hep-1 cells. ***p < 0.001 vs. LO2. **C-D** CCAR1 mRNA and circCCAR1 expression were measured by qRT‒PCR in HCC cells treated with C646 (10 μM) or DMSO for 48 h. **p < 0.01, ***p < 0.001 vs. DMSO. **E** Correlation analysis between EP300 and CCAR1 in TCGA liver hepatocellular carcinoma samples. **F-G** CCAR1 mRNA and circCCAR1 expression were detected after EP300 depletion. ***p < 0.001 vs. sh-NC. **H** EP300 and CCAR1 protein levels in HCC cells after EP300 depletion. **I** The EP300 signals in the CCAR1 promoter were measured using ChIP‒qPCR assays in LO2, HCCLM3 and SK-Hep-1 cells. ***p < 0.001 vs. LO2. **J-K** The H3K27ac signals in the CCAR1 promoter were measured using ChIP‒qPCR assays in HCC cells with EP300 silencing. ***p < 0.001 vs. sh-NC

Histone acetylation plays an important role in gene expression and is involved in regulating the progression of a variety of tumors including HCC [32]. We next explored histone acetylation of circCCAR1 promoter by analyzing Chromatin immunoprecipitation (ChIP)-Seq data from The Encyclopedia of DNA Elements Consortium (ENCODE) database. Enriched histone H3 lysine 27 acetylation (H3K27ac) signals were found in the CCAR1 promoter region (Fig. 4A), showing that histone acetylation may control circCCAR1 or CCAR1 expression. Then, a ChIP assay further indicated that enriched H3K27ac was located in the CCAR1 promoter and was higher in HCC cell lines than in the control LO2 cells (Fig. 4B). Moreover, the histone acetyltransferase (HAT) inhibitor C646 reduced CCAR1 mRNA and circCCAR1 levels (Fig. 4C-D), which further verified that histone acetylation could affect circCCAR1 expression. As E1A binding protein p300 (EP300) is necessary for H3K27ac and positively correlated with CCAR1 expression in TCGA HCC tissues (Fig. 4E), we wondered whether EP300 was implicated in the abnormal expression of circCCAR1 in HCC. To test the hypothesis, the expression of circCCAR1 in HCC cells was detected after EP300 depletion. The inhibition of EP300 significantly decreased CCAR1 mRNA and circCCAR1 expression (Fig. 4F-G). The depletion of EP300 also reduced EP300 and CCAR1 protein levels in HCC cells (Fig. 4H). The results of the ChIP assay suggested that enriched EP300 was located in the CCAR1 promoter and was higher in HCC cell lines than in the control LO2 cells (Fig. 4I). EP300 depletion reduced occupied H3K27ac at the CCAR1 promoter (Fig. 4J-K). These results suggested that EP300-mediated histone acetylation activation increased circCCAR1 expression.

### EIF4A3 promotes the cyclization and nuclear export of circCCAR1

**Fig. 5:**
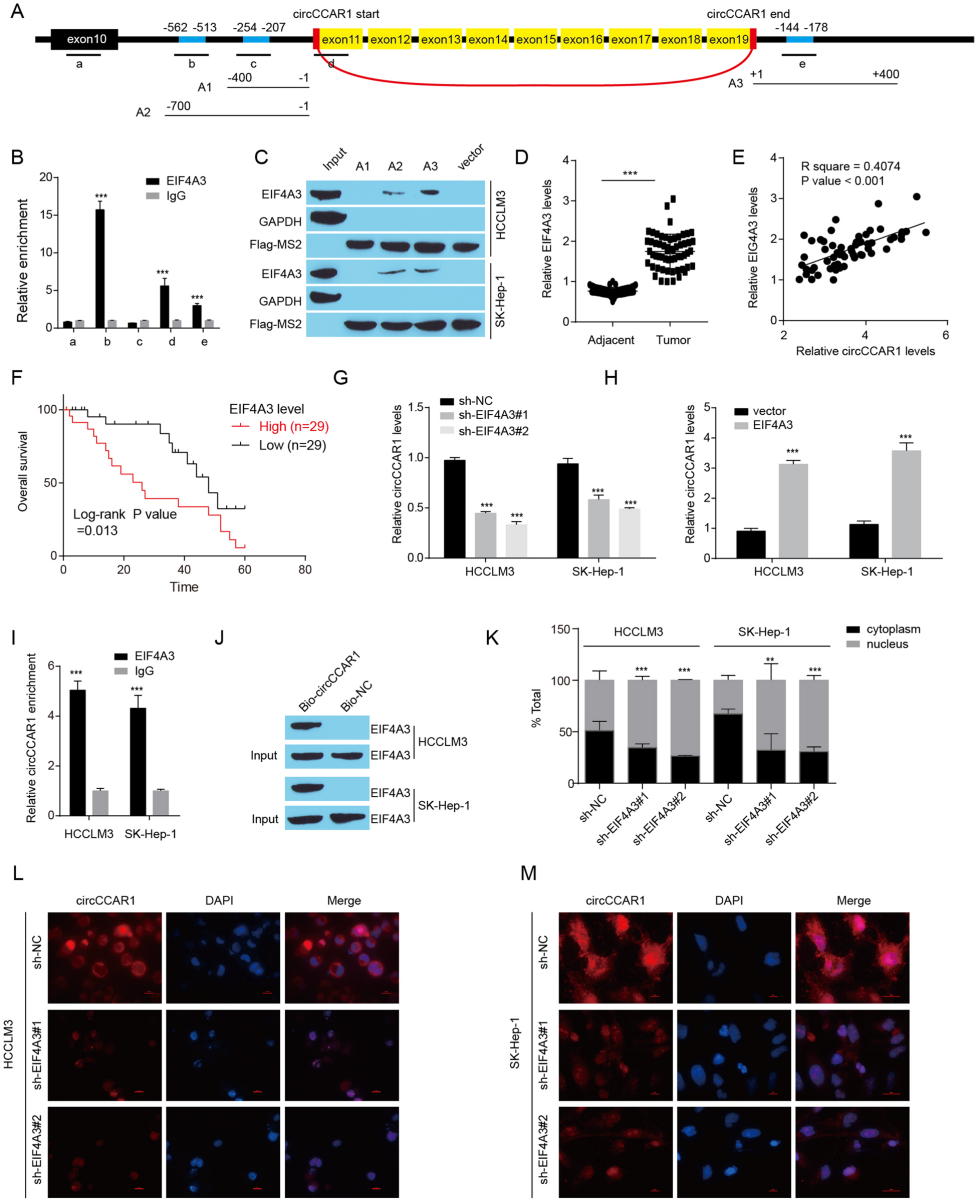
EIF4A3 promotes the cyclization and cytoplasmic export of circCCAR1. **A** Five positions (a-e) in circCCAR1 pre-mRNA were selected to design qPCR primers, and three plasmids (A1-A3) containing EIF4A3 binding sites were constructed to pull down the EIF4A3 protein. **B** The interaction between EIF4A3 and circCCAR1 pre-mRNA was confirmed by RIP assay. ***p < 0.001 vs. IgG. **C** EIF4A3 protein was measured in the MS2-RNA pulldown complex. **D** EIF4A3 levels in HCC tissues and their paracancerous tissues were measured. ***p < 0.001. **E** The relationship between EIF4A3 and circCCAR1 levels in HCC samples was calculated. **F** The association of EIF4A3 expression and overall survival in HCC patients. **G-H** The expression of circCCAR1 in HCCLM3 and SK-Hep-1 cells with EIF4A3 depletion or overexpression. ***p < 0.001 vs. sh-NC or vector. **I** The binding of EIF4A3 and circCCAR1 was confirmed by RIP assay. ***p < 0.001 vs. IgG. **J** EIF4A3 protein level in pulldown assays using biotinylated antisense oligomer. **K-M** Nuclear–cytoplasmic fractionation and FISH-IF assays were used to assess the cytoplasmic export of circCCAR1 in HCC cells after EIF4A3 depletion. **p < 0.01, ***p < 0.001 vs. sh-NC

The interaction between RNA-binding proteins (RBPs) and circRNAs can regulate the generation of circRNAs [33, 34]. We then predicted potential RBPs of circCCAR1 by CircInteractome (https://circinteractome.nia.nih.gov/) and found that eukaryotic translation initiation factor 4A3 (EIF4A3) could bind to the upstream, back-spliced junction site, and downstream flanking sequences of circCCAR1 (Fig. S2A). To verify the specific areas for precursor circCCAR1 to binding EIF4A3, we named a random sequence on exon 10 as a, the back-spliced junction site as d, two upstream putative binding sites as b and c, and one downstream putative binding site as e (Fig. 5A). As indicated by the RNA immunoprecipitation (RIP) assay, we found that EIF4A3 bound to one upstream putative binding site, the back-spliced junction site and one downstream putative binding site (Fig. 5B). Four recombinant plasmids were constructed containing MS2 binding sites and the up- or downstream EIF4A3 binding sites of circCCAR1, which were named A1, A2 and A3, respectively (Fig. 5A). The results of the MS2 RNA pulldown assay affirmed that EIF4A3 bound to the upstream and downstream sequences of circCCAR1 (Fig. 5C). In addition, increased expression of EIF4A3 was found in The Cancer Genome Atlas (TCGA) and Clinical Proteomic Tumor Analysis Consortium (CPTAC) HCC samples when compared with normal samples (Fig. S2B-C). Increased EIF4A3 levels were also found in HCC samples, and there was a positive correlation between circCCAR1 and EIF4A3 expression in HCC samples in-house (Fig. 5D-E). HCC patients with a high level of EIF4A3 had a short overall survival time (Fig. 5F, Fig. S2D). EIF4A3 overexpression increased the circCCAR1 level in HCC cells, and EIF4A3 depletion reduced the circCCAR1 level in HCC cells (Fig. 5G-H). However, the change in EIF4A3 did not affect the mRNA and protein levels of CCAR1 (Fig. S2E-G).

Notably, EIF4A3 was also predicted to bind mature circCCAR1 by CircInteractome databases (Fig. S2H). To verify the interaction between EIF4A3 protein and circCCAR1, RIP and biotinylated RNA pull-down assays were executed. RIP assays indicated that circCCAR1 could be pulled down by anti-EIF4A3 antibodies (Fig. 5I) and biotinylated RNA pull-down assay indicated that EIF4A3 protein was enriched in the circCCAR1 probes complex (Fig. 5J). These data showed the binding between circCCAR1 and EIF4A3 protein in HCC cells. In view of the fact that the interaction between RBPs and circRNAs may alter the stability of circRNAs or RBPs, we next detected the stability of circCCAR1 in EIF4A3 depletion HCC cells and EIF4A3 mRNA or protein levels in circCCAR1 overexpression or depletion HCC cells. However, EIF4A3 knockdown did not affect the stability of circCCAR1 (Fig. S2I-J). CircCCAR1 also did not affect EIF4A3 mRNA or protein levels (Fig. S2K-L). Excepting RNA splicing, EIF4A3 reportedly promotes the nuclear export of spliced RNA. Here, we found a clear reduction in cytoplasmic circCCAR1 levels in HCC cells upon EIF4A3 knockdown (Fig. 5K-M). Moreover, EIF4A3 depletion suppressed the growth, migration, and invasion of HCC cells (Fig. S3), while circCCAR1 overexpression rescued these inhibitory effects induced by EIF4A3 knockdown. In general, these data suggested that EIF4A3 regulates the biogenesis and nuclear export of circCCAR1.

### WTAP-mediated m6A modification enhanced circCCAR1 stability via IGF2BP3

**Fig. 6:**
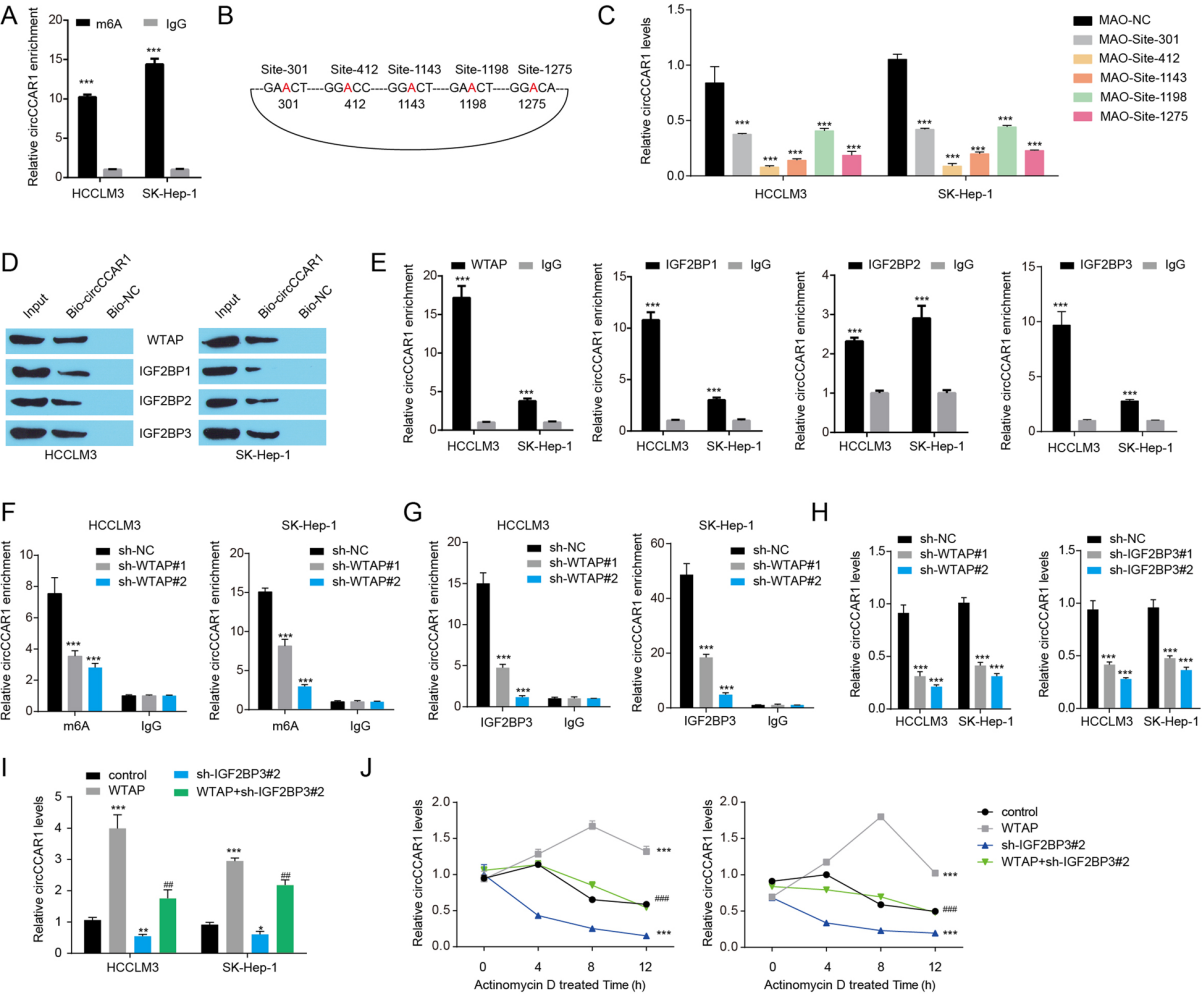
WTAP-mediated m6A modification enhanced circCCAR1 stability via IGF2BP3. **A** m^6^A enrichment in circCCAR1. ***p < 0.001 vs. IgG. **B** Diagram showing the position of m^6^A motifs with a high combined score within circCCAR1. **C** CircCCAR1 levels in HCC cells after treatment with MAO-circCCAR1 or MAO-NC. ***p < 0.001 vs. MAO-NC. **D** WTAP and IGFBPs levels in pulldown assays using a biotinylated antisense oligomer targeting the junction of circCCAR1. **E** The binding of IGF2BPs and circCCAR1 was confirmed by RIP assay. ***p < 0.001 vs. IgG. **F** The m^6^A enrichment in circCCAR1 in HCC cells after WTAP knockdown. ***p < 0.001 vs. sh-NC. **G** IGF2BP3 enrichment in circCCAR1 in HCC cells after WTAP knockdown. ***p < 0.001 vs. sh-NC. **H** CircCCAR1 levels in HCC cells after WTAP knockdown or IGF2BP3 knockdown. ***p < 0.001 vs. sh-NC. **I** CircCCAR1 levels in HCC cells after WTAP overexpression or IGF2BP3 knockdown. *p < 0.05, **p < 0.01, ***p < 0.001 vs. control; ##*p* < 0.01, ###*p* < 0.001 vs. sh-IGF2BP3#2. **J** Stability of circCCAR1 in HCC cells after WTAP overexpression or IGF2BP3 knockdown with actinomycin D treatment. ***p < 0.001 vs. control; ###*p* < 0.001 vs. sh-IGF2BP3#2

N6-methyladenosine (m6A) is a general modification of mRNAs and ncRNAs that influences the splicing, export, translation, and stability of RNAs [25, 35]. To test whether circCCAR1 is modulated by m6A, we used sequence-based RNA adenosine methylation site predictor (SRAMP) to predict potential m6A sites in circCCAR1 and found five m6A sites with a high confidence threshold in circCCAR1 (Fig. S4A). Consistently, MeRIP-qPCR confirmed the enrichment of m6A in circCCAR1 (Fig. 6A). M6A can be introduced by m6A writer, wiped by m6A erasers and recognized by readers. Thus, the potential interaction between circCCAR1 and common m6A writer, erasers and readers proteins was predicted by using the CircInteractome database. We found that the m6A writer WTAP and the m6A readers IGF2BP1, IGF2BP2, and IGF2BP3 were potential CBPs for circCCAR1 (Fig. S4B). Using sequence-specific morpholino antisense oligos (MAOs) targeting the m6A site of circCCAR1, we found that HCC cells treated with MAO targeting these five sites of circCCAR1 exhibited a lower circCCAR1 level (Fig. 6B-C), indicating that these five sites of circCCAR1 were important for circCCAR1 expression. Next, RIP and biotinylated RNA pull-down assays were executed to verify the potential interaction between circCCAR1 and WTAP, IGF2BP1, IGF2BP2 and IGF2BP3. The results indicated that all these four proteins were found to be enriched in the circCCAR1 complex (Fig. 6D-E). Moreover, IGF2BP2 and IGF2BP3 proteins were increased, while IGF2BP1 proteins were decreased in CPTAC HCC samples (Fig. S4C). HCC patients with high IGF2BP2 or IGF2BP3 expression had a poor prognosis (Fig. S4D-F). Subsequently, we focused on exploring the function of IGF2BP3 in the dysregulation of circCCAR1 in HCC. We then explored the potential mechanism for WTAP-m6A-IGF2BP3 axis in the dysregulation of circCCAR1 in HCC. WTAP knockdown drastically decreased the m6A modification of circCCAR1 and the binding of IGF2BP3 to circCCAR1 (Fig. 6F-G), indicating that the binding of IGF2BP3 to circCCAR1 was dependent on WTAP-mediated m6A modification. WTAP knockdown or IGF2BP3 knockdown decreased the level of circCCAR1 in HCC cells (Fig. 6H, Fig. S4G-H). WTAP overexpression enhanced circCCAR1 levels, while IGF2BP3 knockdown reversed the increase in circCCAR1 levels induced by WTAP overexpression (Fig. 6I), indicating that WTAP-m6A-IGF2BP3 axis positively regulate the expression of circCCAR1. Moreover, the stability of circCCAR1 was increased by WTAP overexpression and was decreased by IGF2BP3 knockdown (Fig. 6J). IGF2BP3 knockdown rescued the effect of WTAP overexpression on the stability of circCCAR1. These results indicated that WTAP-m6A-IGF2BP3 axis regulates the expression of circCCAR1 by affecting its stability. In additions, WTAP or IGF2BP3 knockdown inhibited the growth, migration and invasion of HCC cells (Fig. S5), while circCCAR1 overexpression reversed these effects induced by WTAP or IGF2BP3 knockdown, indicating that WTAP-m6A-IGF2BP3 axis induced the upregulation of circCCAR1 was related to the growth, migration and invasion of HCC. In summary, WTAP-mediated m6A modification enhanced circCCAR1 stability via IGF2BP3.

### CircCCAR1 acted as a sponge for miR-127-5p to upregulate its target WTAP

By accident, we found that circCCAR1 overexpression increased the expression of WTAP mRNA, while circCCAR1 knockdown decreased the level of WTAP mRNA in HCC cells (Fig. S6A). We then explored the potential mechanisms between circCCAR1 and WTAP. It had been reported that circRNAs function as miRNA sponges to regulate its targeted mRNA expression [14]. Thus, we performed bioinformatic analysis to predict the potential binding miRNAs of circCCAR1 in the CircInteractome and the potential binding miRNAs of WTAP in TargetScan databases (http://www.targetscan.org). Total 3 miRNAs (miR-127-5p, miR-513a-3p and miR-548c-3p) were overlapped (Fig. S6B). These 3 miRNAs level was found to be significantly increased after circCCAR1 depletion in both HCCLM3 and SK-hep-1 cells (Fig. S6C-D), and miR-127-5p was pitched on for its highest fold change. In addition, miR-127-5p expression levels were significantly decreased in tumor tissues of HCC patients compared with adjacent normal tissues (Fig. S6E) and miR-127-5p expressions in HCC tissues had a negative correlation with circCCAR1 levels (Fig. S6F). Similarly, WTAP expression levels were significantly increased (Fig. S6G) and showed a negative correlation with miR-127-5p levels in tumor tissues of HCC patients (Fig. S6H). These results indicated that circCCAR1-mediated upregulation of WTAP could be through targeting miR-127-5p.

We further tested the interaction between circCCAR1 and miR-127-5p in HCC cells. Through bioinformatics prediction using CircInteractome database, two potential targeting sites were predicted in circCCAR1 and the binding sites between circCCAR1 and miR-127-5p were shown (Fig. S6I). PRL-TK-pMIR-Luc vector including wild type (WT) or mutant (Mut) circCCAR1 fragments were co-transfected with miR-127-5p mimics or miR mimic NC into HCCLM3 and SK-hep-1 cells. The results of luciferase reporter assay showed that miR-127-5p mimics signally decreased the luciferase activity of WT circCCAR1 (Fig. S6J). MiR-127-5p mimics mildly but statistically decreased the luciferase activity of MUT1 circCCAR1 and MUT2 circCCAR1, but exerted no effect on the luciferase activity of MUT1 + MUT2 circCCAR1, indicating that these two sites synergistically help to the binding between circCCAR1 and miR-127-5p. Besides, RIP assay indicated that the enrichment of endogenous circCCAR1 and miR-127-5p pulled-down from Ago2-expressed HCCLM3 and SK-hep-1 cells was markedly elevated in Ago2 pellet as compared with the input control (Fig. S6K). Furthermore, the results of functional experiments showed that miR-127-5p mimics inhibited the growth, migration and invasion of HCC cells (Fig. S6L-N). MiR-127-5p mimics reversed the change of growth, migration and invasion of HCC cells induced by circCCAR1 overexpression. Therefore, circCCAR1 may affect the growth, migration and invasion of HCC cells partly by acting as a sponge for miR-127-5p.

The interaction between WTAP 3’untranslated region (3’UTR) and miR-127-5p in HCC cells was then verified. According to TargetScan database, we found that miR-127-5p could bind to the 3’UTR region of WTAP. The putative binding sequence between miR-127-5p and WTAP was shown (Fig. S7A). Next, luciferase reporter assays were performed to verify this interaction. The results showed that cotransfection of miR-127-5p mimics could strongly reduce the activity of a luciferase reporter carrying the wild-type WTAP 3’UTR compared to miR mimic NC. Inversely, the mutated luciferase reporter was unaffected by over-expression of miR-127-5p (Fig. S7B). The results of qRT-PCR and Western blotting assays demonstrated that miR-127-5p mimics could decrease the level of WTAP mRNA and protein (Fig. S7C-D), indicating that miR-127-5p inhibited the expression of WTAP by targeting its 3’UTR. Besides, the effects of miR-127-5p/WTAP axis on the phenotypes of HCC cells were also analyzed. Through conducting CCK8 assay, Transwell migration and invasion assay, we found that WTAP overexpression promoted the growth, migration and invasion of HCC cells (Fig. S7E-G). WTAP overexpression rescued the change of growth, migration and invasion of HCC cells reduced by miR-127-5p mimics, displayed that miR-127-5p could regulate the growth, migration, invasion of HCC partly through targeting WTAP. In general, circCCAR1 affected the growth, migration, invasion of HCC via the miR-127-5p/WTAP axis.

### hnRNPA2B1-mediated circCCAR1 packaging into exosomes

**Fig. 7:**
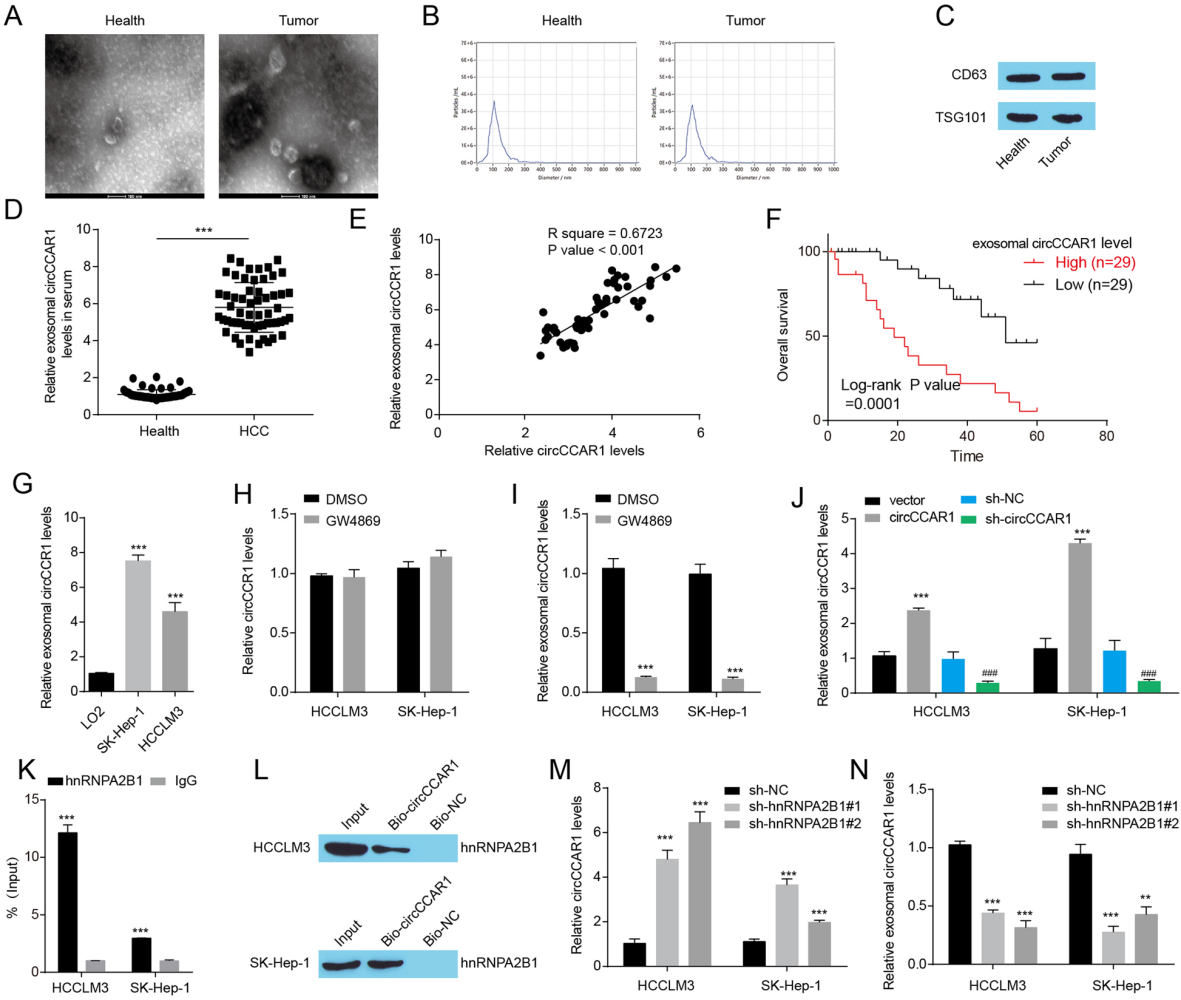
Exosomal circCCAR1 is increased in HCC patients. **A** Transmission electron microscopy detected the exosomes. **B** NanoSight particle tracking analysis of the size distributions and number of exosomes. **C** The levels of exosome markers (CD63 and TSG101) in purified exosomes. **D** The level of circCCAR1 in exosomes from the serum of HCC patients and healthy donors. ***p < 0.001. **E** The correlation between exosomal circCCAR1 in serum and circCCAR1 in tumor samples was assessed by Spearman correlation analysis. **F** The association of exosomal circCCAR1 levels and overall survival in HCC patients. **G** The level of circCCAR1 in exosomes in culture medium from LO2 cells and HCC cells. ***p < 0.001 vs. LO2. **H-I** The level of circCCAR1 in HCC cells or exosomes isolated from the supernatants of HCC cells treated with GW4869. ***p < 0.001 vs. DMSO. **J** The level of circCCAR1 in exosomes in HCC cells after circCCAR1 overexpression or depletion. ***p < 0.001 vs. vector; ###*p* < 0.001 vs. sh-NC. **K** The binding of circCCAR1 and hnRNPA2B1 was confirmed by RIP assay. ***p < 0.001 vs. IgG. **L** The binding of circCCAR1 and hnRNPA2B1 was confirmed by RNA pulldown assay. **M** The relative circCCAR1 expression in HCC cells after hnRNPA2B1 knockdown. **N** The relative circCCAR1 expression in exosomes after hnRNPA2B1 knockdown. ***p < 0.001 vs. sh-NC

Previous research has shown that circRNAs packaged into exosomes have an important function in malignancies [36]. Increased circCCAR1 expression was found in serum from HCC patients when compared with normal individuals by microarray analysis (Fig. 1A). Subsequently, we purified exosomes from the serum of 58 HCC patients and 58 healthy donors. Classic characteristics of exosomes were presented in purified exosomes: diameter ranging from 50 to 100 nm, a cup-shaped morphology, and expressed marker proteins CD63 and TSG101 (Fig. 7A-C), indicating that we successfully isolated exosomes from serum in HCC patients. A higher level of circCCAR1 in exosomes was detected in serum from HCC patients (Fig. 7D). The levels of circCCAR1 in tumor tissues were positively related to the levels of exosomal circCCAR1 in serum from HCC patients (Fig. 7E). Exosomal circCCAR1 in serum could be regarded as a prognostic indicator (Fig. 7F). In addition, increased exosomal circCCAR1 levels were observed in the cell culture medium from HCC cells compared with LO2 cells (Fig. 7G). Blocking exosome formation by a pharmacological inhibitor of neutral sphingomyelinase-2 (nSMase) GW4869 significantly inhibited the levels of exosomal circCCAR1, with no effect on the levels of circCCAR1 in HCC cells (Fig. 7H-I), thus confirming the existence of circCCAR1 in exosomes. Furthermore, circCCAR1 overexpression in HCC cells enhanced the levels of exosomal circCCAR1, and circCCAR1 depletion reduced the levels of exosomal circCCAR1 (Fig. 7J). In short, these results indicated that circCCAR1 could be packaged into exosomes.

We then explored the potential mechanism by which circCCAR1 is encapsulated into exosomes. As hnRNPA2B1 is reported to transport various RNAs into exosomes [37], the interaction between circCCAR1 and hnRNPA2B1 was evaluate and found that circCCAR1 could bind to hnRNPA2B1 by RIP and RNA pull-down assays in HCC cells (Fig. 7K-L). In addition, circCCAR1 expression was enhanced in hnRNPA2B1-depleted HCC cells and reduced in exosomes secreted by hnRNPA2B1-depleted HCC cells (Fig. 7M-N). In summary, our data suggested that circCCAR1 could be packaged into exosomes in a hnRNPA2B1-dependent manner.

### Exosomal circCCAR1 accelerates the exhaustion of antitumor CD8 + T cells

**Fig. 8:**
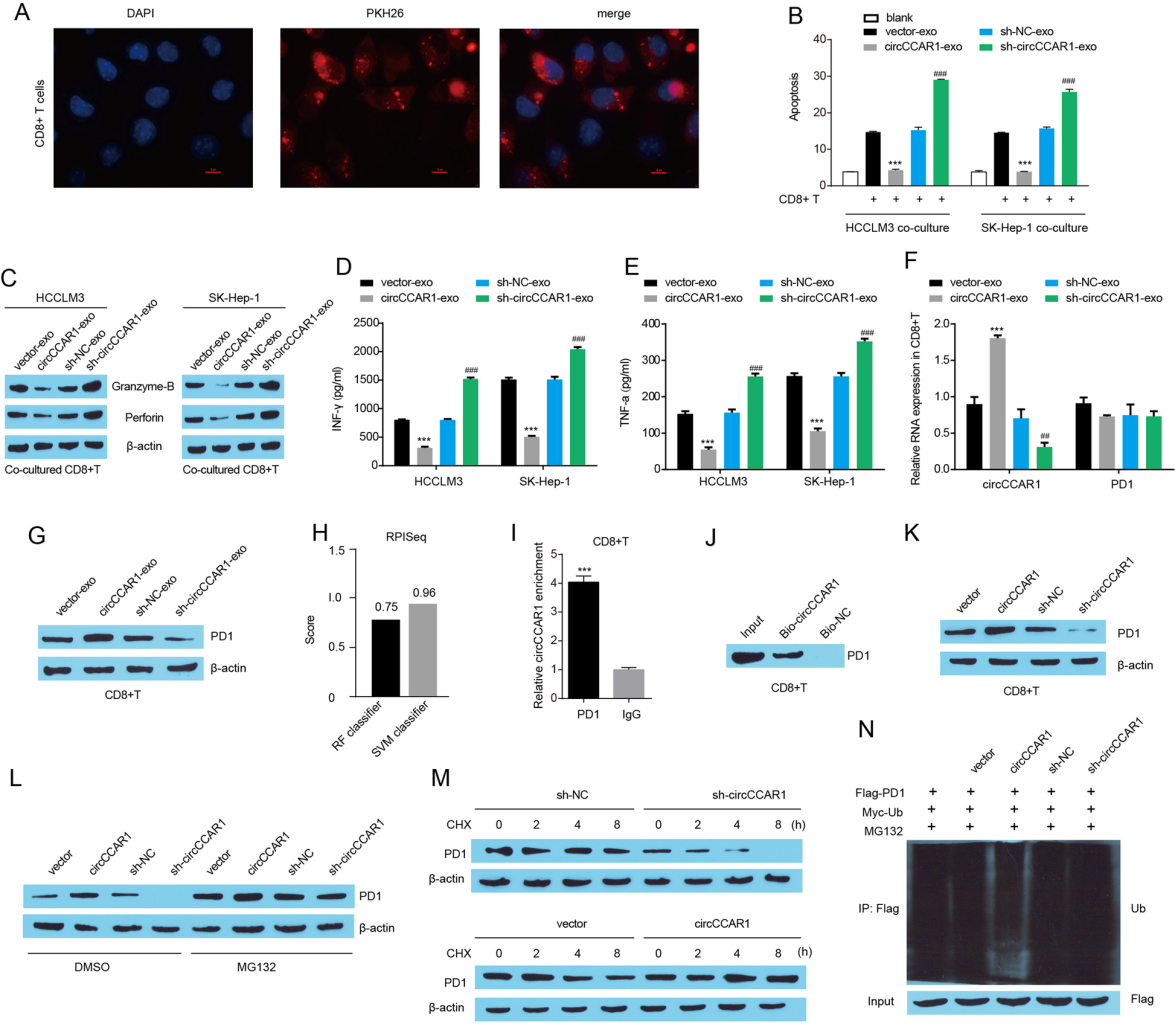
Exosomal circCCAR1 protects HCC cells from CD8^+^ T cells by stabilizing PD-1. **A** Representative images of the internalization of PKH67-labeled HCCLM3 exosomes (red) by CD8 + T cells. **B** CD8 + T-cell-pretreated exosomes were cocultured with HCC cells, and then CD8 + T-cell-mediated elimination of HCC cells was determined by FACS analysis. ***p < 0.001 vs. vector-exo; ###*p* < 0.001 vs. sh-NC-exo. **C** Perforin and granzyme-B levels in CD8 + T cells. **D-E** Secreted IFN-γ and TNF-α by CD8 + T cells were measured by ELISA. ***p < 0.001 vs. vector-exo; ###*p* < 0.001 vs. sh-NC-exo. **F** circCCAR1 and PD1 mRNA expression in CD8 + T cells. ***p < 0.001 vs. vector-exo; ##*p* < 0.01 vs. sh-NC-exo. **G** PD1 protein expression in CD8 + T cells. **H** The interaction strength between circCCAR1 and PD1 was determined using the RPISeq program. **I** The binding of circCCAR1 and PD1 was confirmed by RIP assay. ***p < 0.001. **J** PD1 levels in pulldown assays using a biotinylated antisense oligomer targeting the junction of circCCAR1 in CD8 + T cells. **K** PD1 expression in CD8 + T cells after circCCAR1 depletion or overexpression. **L** PD1 protein levels in CD8 + T cells treated with MG132 after circCCAR1 depletion or overexpression. **M** Stability analysis of PD1 protein in CD8 + T cells treated with CHX after circCCAR1 depletion or overexpression. **N** Ubiquitination assay of PD1 in CD8 + T cells

Studies have shown that exosomes secreted by tumor cells can regulate tumor progression by regulating immune response [38–40]. Tumor-infiltrating CD8 + T cells are considered the most important antitumor cell types, thus, we investigated the effect of exosomal circCCAR1 on CD8 + T cells. Exosomes were isolated from the cultural supernatant of HCC cells and incubated with activated CD8 + T cells. For exosome tracking**,** HCCLM3-derived exosomes were labeled with the red fluorescent dye PKH26 and we found that exosomes were efficiently absorbed by activated CD8 + T cells (Fig. 8A). To confirm whether circCCAR1 participates in CD8 + T cell proliferation, we examined CD8 + T cell activity in vitro. Indeed, expression of the cell proliferation marker Ki-67 after CD8 T cell activation was attenuated in exosomal circCCAR1 treated CD8 + T cells (Fig. S8A), indicating that the co-culture of exosomal circCCAR1 and activated CD8 + T cells inhibited the growth of activated CD8 T cells. Moreover, TUNEL assay displayed that exosomal circCCAR1 treatment enhanced the apoptosis of activated CD8 T cells (Fig. S8B). A T-cell-mediated killing assay was performed to assess the cytotoxicity of activated CD8 + T cells. CD8 + T cells cocultured with exosomes from circCCAR1-overexpressing HCC cells had a weaker cytotoxicity to HCC cells (Fig. 8B). The coculture of exosomal circCCAR1 reduced the protein levels of perforin and granzyme B in activated CD8 + T cells and inhibited the secretion of IFN-γ and TNF-α in cultural supernatant of activated CD8 + T cells (Fig. 8C-E). Moreover, we also detected the expression of common markers in cell surface of CD8 + T cell exhaustion (for example, PD1, LAG3, TIM3, and TIGIT) by flow cytometry. We found that exosomes derived from circCCAR1 overexpressed HCCLM3 cells increased the level of LAG3, PD1, TIM3, and TIGIT in cell surface of CD8 + T cells (Fig. S8C-F). All these results indicated that exosomal circCCAR1 promoted the dysfunction of activated CD8 + T cells by inhibiting its proliferation, promoting its apoptosis, reducing its cytotoxicity and secretion of cytokines, and increasing LAG3, PD1, TIM3, and TIGIT expression in cell surface of CD8 + T cells. In addition, we also found that exosomes from circCCAR1-knockdown HCC cells played the opposite role, indicating that reducing the level of circCCAR1 in exosomes could enhance the function of CD8 + T cells.

PD1 is highly expressed in exhausted CD8 + T cells, and targeting PD1 could enhance CD8 + effector T-cell function and inhibit the malignant progression of tumors [41, 42]. Here, we found that among these four common markers of CD8 + T cell exhaustion, PD1 levels in cell surface of CD8 + T cells showed a highest fold change when co-cultured with exosomal circCCAR1 (Fig. S8C-F). We then focused on exploring the underlying mechanisms in the upregulation of PD1 in cell surface of CD8 + T cells induced by exosomal circCCAR1. Exosomes from HCCLM3 cells overexpressing circCCAR1 increased circCCAR1 levels and PD1 protein expression in CD8 + T cells (Fig. 8F-G). Exosomes from HCCLM3 cells with circCCAR1 knockdown performed the opposite function. However, exosomes from HCCLM3 cells with circCCAR1 overexpression or knockdown did not affect PD1 mRNA expression, suggesting that exosomal circCCAR1 may regulate PD1 levels at the posttranscriptional level. The RNA–Protein Interaction Prediction (RPISeq) tool was used to analyze the potential interaction between circCCAR1 and PD1 protein and the results showed that the scores of the RF classifier and SVM classifier were no less than 0.7 (Fig. 8H), implying high binding potential between circCCAR1 and PD1 protein. Then, RIP and biotinylated RNA pull-down assays further carried out and the results verified the interaction between circCCAR1 and PD1 in CD8 + T cells (Fig. 8I-J). The depletion of circCCAR1 decreased PD1 protein levels, and overexpression of circCCAR1 increased PD1 protein levels in activated CD8 + T cells (Fig. 8K). We then explored whether circCCAR1 regulates PD1 degradation through proteolysis in CD8 + T cells. The proteasome inhibitor MG132 rescued the change in PD1 protein expression induced by circCCAR1 depletion or overexpression in CD8 + T cells (Fig. 8L), suggesting that circCCAR1 prevents proteasomal degradation of PD1 proteins by binding PD1 proteins. In addition, circCCAR1 depletion significantly shortened the half-life of the PD1 protein, and circCCAR1 overexpression greatly prolonged the half-life of the PD1 protein in CD8 + T cells (Fig. 8M). Through a ubiquitination assay, we found that circCCAR1 overexpression greatly inhibited the ubiquitination of PD1 proteins, whereas circCCAR1 depletion increased the ubiquitination of PD1 proteins (Fig. 8N). Collectively, these results demonstrated that circCCAR1 interacted with PD1 to prevent its degradation by reducing the ubiquitination of PD1 proteins in activated CD8 + T cells.

To investigate the effect of the exosomal circCCAR1-PD1 axis on CD8 + T cell-mediated cytotoxicity, HCC cells were cocultured with activated CD8^+^ T cells and the apoptosis of HCC cells was measured. CircCCAR1 depletion in HCC cells promoted the apoptosis of HCC cells mediated by CD8 + T cells (Fig. S9A-B). Overexpression of PD-1 in CD8 + T cells reduced the effect of circCCAR1 depletion on the resistance of HCC cells to CD8 + T cells. Moreover, pretreatment with GW4869 in HCC cells enhanced the percentage of apoptotic HCC cells induced by CD8 + T cells (Fig. S9C-D). PD-1 overexpression reversed the change in GW4869 on CD8 + T-cell-induced apoptosis of HCC cells. Moreover, PD-1 overexpression in CD8 + T cells rescued the apoptosis of HCC cells induced by CD8 + T cells pretreated with exosomes from circCCAR1-depleted HCC cells (Fig. S9E-F). Therefore, exosomal circCCAR1 protects HCC from CD8 + T cells by stabilizing PD-1.

### CCAR1 enhanced PD-L1 expression by interacting with β-catenin

CCAR1 reportedly interacts with β-catenin and enhances the ability of β-catenin to activate the transcription of target genes [43]. As PD-L1 is reportedly a target for β-catenin [44], we inferred that CCAR1 may promote transcriptional expression of PD-L1 by binding β-catenin. CCAR1 was increased in TCGA HCC samples and was positively correlated with tumor grade, individual cancer stages and nodal metastasis status (Fig. S10A-C). HCC patients with high CCAR1 levels had an unfavorable prognosis (Fig. S10D). The protein level of CCAR1 was increased in CPTAC HCC samples compared with normal samples (Fig. S10E). A high level of CCAR1 mRNA and protein was also found in HCC cell lines (Fig. S10F-G). Coimmunoprecipitation was first conducted with extracts of HCCLM3 or SK-Hep-1 cells to verify the interaction between CCAR1 and β-catenin. CCAR1 was precipitated by anti-β-catenin antibodies, and β-catenin was precipitated by anti-CCAR1 antibodies (Fig. S10H-I), indicating the endogenous interaction between CCAR1 and β-catenin. To further understand the effects of CCAR1/β-catenin on PD-L1 transcription, we subcloned the promoter sequence of PD-L1 into p-GL3-Basic vectors and cotransfected them and shRNA for CCAR1 or β-catenin into HCC cells. Knockdown of CCAR1 or β-catenin significantly reduced luciferase activity (Fig. S10J), indicating that CCAR1 or β-catenin knockdown reduced PD-L1 promoter activity. ChIP analysis showed an enrichment of CCAR1 and β-catenin at the PD-L1 promoter (Fig. S10K-L). β-catenin knockdown greatly eliminated occupancy of the PD-L1 promoter by β-catenin and CCAR1, whereas CCAR1 knockdown greatly eliminated occupancy of the PD-L1 promoter by CCAR1. As CCAR1 knockdown had no effect on β-catenin protein expression in HCC cells (Fig. S10M), we concluded that CCAR1 may contribute to the stable occupancy of the PD-L1 promoter by β-catenin. Moreover, the depletion of CCAR1 or β-catenin inhibited PD-L1 mRNA and protein expression in HCC cells (Fig. S10M-N). Therefore, our results showed that CCAR1 regulated PD-L1 expression by interacting with β-catenin.

### CircCCAR1 promotes the resistance of HCC to anti-PD1 therapy

**Fig. 9:**
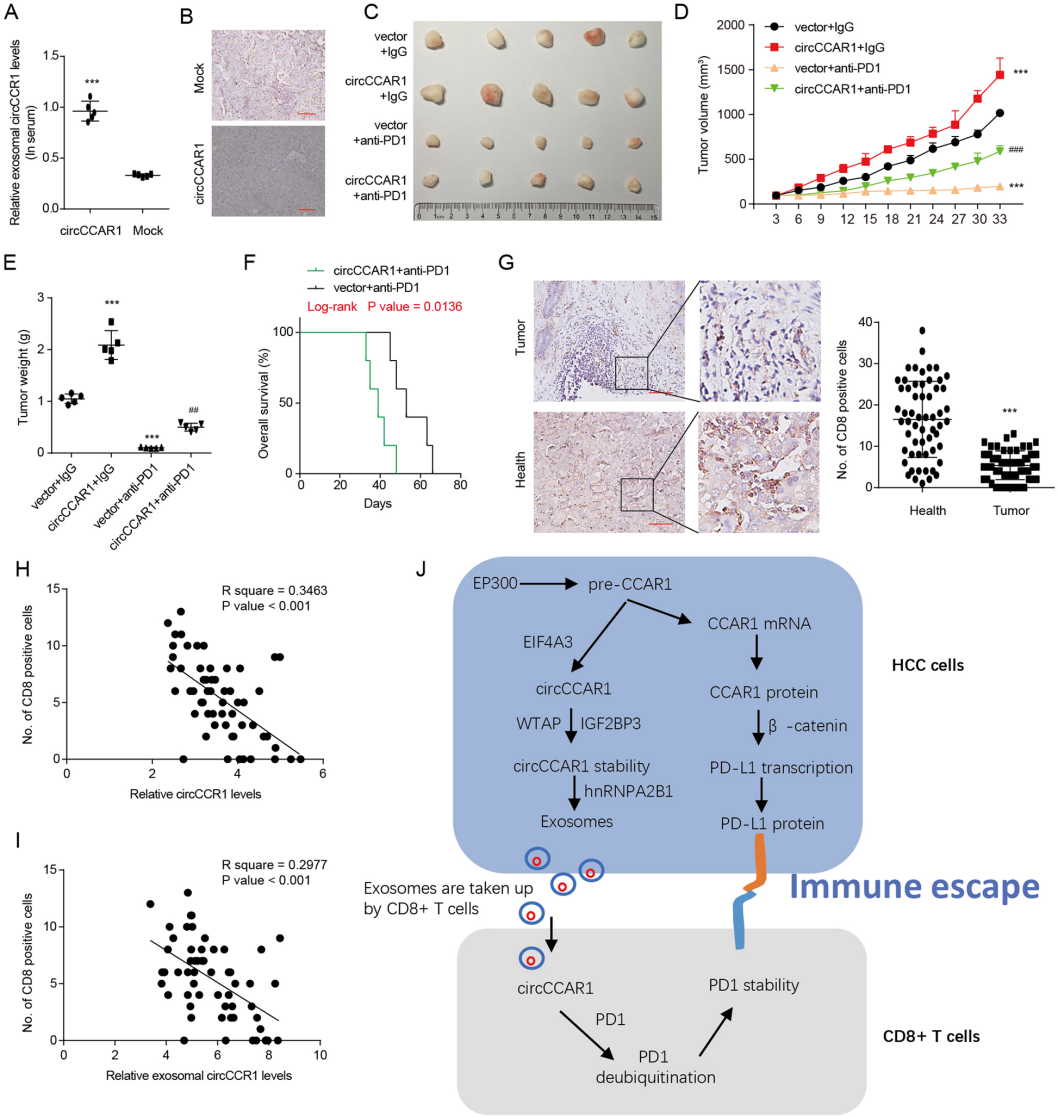
CircCCAR1 promotes the resistance of HCC to anti-PD1 therapy. **A** Exosomal circCCAR1 levels in the serum from HuNSG mice. ***p < 0.001. **B** CD8-positive cells in HCCLM3-circCCAR1 or HCCLM3-mock cell-derived xenografts were detected by CD8 staining. **C-E** The tumor volume and weight. **F** The survival curves for mice with xenografts. **G** CD8 in tissues from HCC patients was analyzed by CD8 staining. ***p < 0.001. **H** The relationship between circCCAR1 and infiltrated CD8-positive cells in the HCC tissues was calculated. **I** The relationship between exosomal circCCAR1 in serum and CD8-positive cell numbers in HCC tissues was also calculated. **J** Model of the circCCAR1-mediated immunosuppressive effect in HCC

To explore the role of circCCAR1 in anti-PD1 therapy, a HuNSG mouse xenograft model was constructed. Exosomal circCCAR1 in the peripheral blood from HuNSG mice bearing xenografts derived from circCCAR1-overexpressing HCCLM3 cells was increased compared with that in the mock-treated mice (Fig. 9A). The results of CD8 IHC staining indicated that more CD8 + T cells were observed in mock cell-derived tissues than in circCCAR1-overexpressing cell-derived tissues (Fig. 9B). To confirm the function of circCCAR1 in resistance to anti-PD1 therapy, Opdivo was injected into HuNSG mice which received circCCAR1-overexpressing HCCLM3 cells or mock cells. The xenograft mice with high circCCAR1 expression were resistant to anti-PD1 therapy and showed a shorter survival time (Fig. 9C-F). Our data suggested that circCCAR1 participated in the anti-PD1 therapy resistance of HCC.

Moreover, the relationship between circCCAR1 and the infiltration of CD8 + T cells in HCC patients was further verified. Results of CD8 IHC staining indicated that the infiltration of CD8 + T cells in the HCC tissues was decreased when compared with that in the adjacent normal tissues (Fig. 9G). Both circCCAR1 and exosomal circCCAR1 levels in HCC tissues were negatively related to CD8 + T-cell frequency (Fig. 9H-I), indicating that circCCAR1 may affect the infiltration of CD8 + T cells in tumor tissues of HCC patients.

## Discussion

CircRNAs are abnormally expressed in many types of tumors and control the progression of tumor malignancy [34, 45]. CircRNAs are related to tumor proliferation, apoptosis, metastasis, metabolism, immunosuppression, and chemotherapy resistance. Nevertheless, the biological functions and mechanisms of circRNA in various tumors remain unclear. In this study, we identified circCCAR1, which was increased in HCC tissues and enriched in serum exosomes of HCC patients. CircCCAR1 facilitated the growth and metastasis of HCC cells in vitro and in vivo. Our results further confirmed the tumorigenic function of circCCAR1.

Impaired antitumor immunity mediated by CD8 + T-cell dysfunction is a hallmark of cancer in the tumor microenvironment (TME) [6]. Long-term persistence of tumor antigens and/or suppression of the TME pushes antitumor effector CD8 + T cells into a state of impaired function known as "T-cell exhaustion" [7]. Exhausted CD8 + T cells highly express inhibitory receptors, such as PD1, and show weakened cytotoxic function and decreased capacity to generate effector cytokines. In this study, we found that CD8 + T cells incubated with exosomes from circCCAR1 knockdown HCC cells had stronger cytotoxicity to HCC cells and produced more perforin, granzyme B, IFN-γ and TNF-α. We found that circCCAR1 prevented PD1 from ubiquitination-mediated degradation by directly binding PD1. PD1 overexpression in CD8 + T cells decreased the tumor-killing ability of CD8 + T cells induced by exosomes from circCCAR1-knockdown HCC cells. Thus, exosomal circCCAR1 can be used as a promising target for rescuing T-cell exhaustion and improving the effect of antitumor immunotherapy.

Signal exchange between cells contributes to the immune response of cancer, in which exosomes are an important means of communication between cells [36, 46]. Abnormal expression of exosomal circRNAs is found in the peripheral blood of cancer patients [47]. Notably, exosomal circRNA is related to the immune avoidance process of tumors. Chen et al. indicated that exosomal circUSP7 secreted by non-small cell lung cancer cells promoted CD8 + T-cell exhaustion and anti-PD1 resistance [31]. Exosomal circTRPS1-secreting tumor cells accelerate malignant processes and CD8 + T-cell dysfunction in bladder cancer [40]. Here, we suggest that exosome-derived circCCAR1 is taken in by CD8 + T cells, which further increases the stability of PD1, causes CD8 + T-cell exhaustion and leads to tumor immunosuppression. Notably, HCC cells with high circCCAR1 expression were resistant to anti-PD1 treatment. Moreover, we found that the mRNA and protein levels of CCAR1, the host gene of circCCAR1, were increased in HCC. The increased CCAR1 promoted PD-L1 expression by interacting with β-catenin, further decreasing the immune response of HCC. Thus, exosomal circCCAR1 secreted by HCC cells promotes resistance to anti-PD1 therapy by accelerating CD8 + T-cell dysfunction, and targeting exosomal circCCAR1 may be a potential therapeutic strategy for HCC patients.

Our results suggest that circCCAR1 accelerates tumor progression and immune evasion in HCC patients. A higher level of circCCAR1 in exosomes was detected in serum from HCC patients. Exosomal circCCAR1 in serum could be regarded as a diagnostic and prognostic indicator in HCC patients. HCC cells with high circCCAR1 levels were resistant to anti-PD1 treatment. These results indicated that exosomal circCCAR1 expression can be used as a novel and effective marker for anti-PD1 therapy in HCC.

The survival time of tumor patients is influenced by CD8 + T-cell infiltration. PD1 acts as an inhibitory receptor and is generally expressed in T cells, NK cells, and B cells [48, 49]. High expression of PD1 results in CD8 + T-cell exhaustion, and targeting PD1 could cause CD8 + T-cell activation [50, 51]. Our study indicated that poor prognosis was related to high expression of circCCAR1, exosomal circCCAR1, and CCAR1. The higher expression of exosomal circCCAR1 suppresses the cytotoxicity and the capability of CD8 + T cells to produce TNF-α, IFN-γ, granzyme-B, and perforin by inhibiting ubiquitination degradation of PD1 and promoting CD8 + T-cell exhaustion. Enhanced circCCAR1 expression reduced the treatment effect of anti-PD1 therapy via exosomal circCCAR1. Moreover, we also found that CCAR1 silencing sensitized HCC cells to CD8 + T-mediated cytotoxicity by inhibiting the transcription of PD-L1 by interacting with β-catenin in HCC cells.

## Conclusion

In conclusion, circCCAR1 and CCAR1 are enhanced in HCC and could be prognostic factors for HCC patients. The circCCAR1/miR-127-5p/WTAP positive feedback loop enhances the growth and metastasis of HCC. Exosomal circCCAR1 secreted by HCC cells can be took in by activated CD8 + T cells and promotes the dysfunction of CD8 + T cells by enhancing the stability of the PD1 protein. Moreover, increased CCAR1 protein expression in HCC cells promotes the transcription of PD-L1 by binding β-catenin, which may enhance the resistance for anti-PD1 therapy (Fig. 9J). Therefore, targeting exosomal circCCAR1 or CCAR1 may offer a new strategy to maximize immunotherapeutic efficacy in HCC patients.

## Supplementary Information

**Additional file 1.**

## Data Availability

All data generated or analyzed during this study are included either in this article or in the supplementary information files.

## Abbreviations

CCAR1: Cell division cycle and apoptosis regulator 1
TEM: Transmission electronic microscopy
ELISA: Enzyme linked immunosorbent assay
EP300: Recombinant E1A Binding Protein P300
EIF4A3: Eukaryotic translation initiation factor 4A3
WTAP: WT1 associated protein
IGF2BP3: Insulin like growth factor 2 mRNA binding protein 3
hnRNPA2B1: Heterogeneous nuclear ribonucleoprotein A2/B1
qRT‒PCR: Quantitative real-time polymerase chain reaction
HAT: Histone acetyltransferase
RBPs: RNA-binding proteins
m6A: N6-methyladenosine
TME: Tumor microenvironment
ChIP: Chromatin immunoprecipitation
RIP: RNA binding protein immunoprecipitation
IHC: Immunohistochemistry
FISH: Fluorescence in situ hybridization
ENCODE: Encyclopedia of DNA elements
TCGA: The cancer genome atlas
CPTAC: Clinical proteomic tumor analysis consortium
MAOs: Morpholino antisense oligos
