# Supplementary material for: Exosome-derived circCCAR1 promotes CD8 + T-cell dysfunction and anti-PD1 resistance in hepatocellular carcinoma
Source: Mol Cancer. 2023 Mar 18;22:55. doi: 10.1186/s12943-023-01759-1 (PMC10024440; doi:10.1186/s12943-023-01759-1)
Supplement: Supplementary file 1 — Additional file 1. [file 12943_2023_1759_MOESM1_ESM.docx]

**Supplementary Materials and Methods**

**Bioinformatics analysis**

The GEO database (GSE100206 and GSE100207) was employed to filter the abnormal circRNAs in the serum exosomes of HCC patients. The filter criteria were set to |fold changes| ≥ 2.0 with p values < 0.05.

**RNA fluorescence in situ hybridization (FISH)**

Cells were first made into paraffin sections. After permeabilization, the cells were hybridized with Cy3‐labeled circCCAR1 probes at 37 °C overnight. After counterstained with DAPI, the sections were observed under a confocal laser microscope.

**Cell culture**

The normal human liver cell line (LO2), and human HCC cell line (HCCLM3, SK-Hep-1, SMMC-7721, Huh7, HepG2) were all purchased from the Chinese Cell Bank of the Chinese Academy of Sciences (Shanghai, China). All cells were maintained and stored following the instructions obtained from their providers. Briefly, LO2, SMMC‐7721, HepG2, Huh7, SK-Hep-1, and HCCLM3 cells were cultured in DMEM (Solarbio, Beijing, China) supplemented with 10% fetal bovine serum (Solarbio) and 1% penicillin‒streptomycin (Beyotime). All cell lines were cultured in a humidified incubator with 5% CO_2_ at 37 °C.

**RNase R treatment**

Total RNA of the indicated cells was treated with or without RNase R (6 U/μg, Beyotime) for 10 min at 37 °C. After purification, the expression of circCCAR1 was determined by qRT-PCR.

**Actinomycin D assay**

Cells were exposed to actinomycin D (2 μg/ml, Sigma‒Aldrich) for the indicated times (0, 8, 24, and 48 h). The total RNA of cells was then collected. The expression of circCCAR1 was determined by qRT-PCR.

**Plasmid construction and cell transfection**

The miRNA mimics, miRNA inhibitors and shRNAs targeting circCCAR1, EP300, EIF4A3, WTAP, IGF2BP1, hnRNPA2B1, CCAR1, β-catenin and overexpression plasmids for circCCAR1, EIF4A3, WTAP, PD1, PD-L1 and were synthesized by GenePharma. For transient transfection of plasmids (2 μg/mL), shRNAs (50 nM), miRNA mimics (50 nM), Lipofectamine™ 3000 kit (Thermo Fisher Scientific, Waltham, USA) was used. The sequences of these nucleic acids are listed in Table S2.

**Cell Counting Kit-8 (CCK-8)**

For the cell proliferation assay, 1 × 10^3^ cells were seeded in 100 μL of complete culture media in 96-well plates for various time points. Then, 10 μl CCK‐8 (Beyotime) was supplemented to each well at 37 °C for 1 hour. Then, the absorbance at 450 nm was detected by an automatic microplate reader.

**Colony formation assay**

For the colony formation assay, 1000 cells were inoculated into 6-well plates and cultured at 37°C for 14 days. The cells were then fixed with 4% paraformaldehyde (PFA) and stained with 0.5% crystal violet (Beyotime). The number of cell clone was counted.

**Wound healing assay**

Cells were inoculated in 12-well plates to form cell monolayers. A 200 μL sterile plastic tip was used to create a wound line. After removal of suspended cells, cells were cultured in serum-free DMEM for 24 hours. The width of the scratch was measured with a phase-contrast microscope.

**Transwell assay**

For the migration assay, approximately 1 × 10^5^ HCC cells were seeded in the upper chamber with serum-free DMEM and the lower chamber was filled with culture medium with 10% FBS as an attractant. After 48 hours, the chamber was immersed in 4% paraformaldehyde and stained with 0.1% crystal violet. Cells on the surface of the upper chamber were wiped off and then observed under a microscope. 10 fields were selected to count the cells to reflect cell mobility. For the invasion assay, the insert membranes were precoated with Matrigel (50 mL/well) (BD Biosciences). The other procedure was the same as the migration assay.

**Western blotting**

Total protein was extracted, quantified, separated and transferred onto PVDF membranes (Millipore). Then, the membranes were blocked, incubated overnight at 4 °C with the primary antibodies, incubated with the secondary antibodies for 1 hour, and observed using ECL chemiluminescence reagent (Beyotime). The primary antibodies were anti‐EP300 (ab275378), anti‐CCAR1 (ab70245), anti‐EIF4A3 (ab180573), anti‐WTAP (ab195380), anti‐IGF2BP1 (ab290736), anti‐IGF2BP2 (ab129071), anti‐IGF2BP3 (ab177477), anti‐β‐catenin (ab32572), anti‐granzyme-B (ab134933), anti‐perforin (ab256453), anti‐PD1 (ab237728), anti‐PD-L1 (ab205921), anti‐Ub (ab134953), anti‐β‐actin (ab8226), and anti‐GAPDH (ab8245). All antibodies were obtained from Abcam.

**Protein half-life assays**

HCCLM3 cells with circCCAR1 overexpression or knockdown were individually treated with 20 μg/mL cycloheximide (CHX) or DMSO for 0, 2, 4, and 8 hours. Then, the protein level of PD1 was measured by Western blotting.

**Ubiquitination assay**

HCCLM3 cells with circCCAR1 overexpression or knockdown were transfected with Myc-Ub and Flag-PD1 for 48 hours of incubation. Then, cells were treated with 20 μM MG132 for 8 hours to induce the accumulation of ubiquitinated proteins. The ubiquitinated PD1 protein was immunoprecipitated by using Flag affinity beads, and endogenous ubiquitination levels of PD1 were detected by the anti-Myc antibody.

**Luciferase reporter assay**

Dual-luciferase reporter vectors carrying the wild type (WT) fragments (Position: 500-1300) of circCCAR1 (PRL-TK-pMIR-circCCAR1) or its mutant (MUT) fragments, carrying the WT fragments of WTAP 3’untranslated region (3’UTR) containing predicted target sites (PRL-TK-pMIR-WTAP 3’UTR) or its MUT fragments were constructed. HCCLM3 and SK-hep-1 cells were co-transfected with renilla luciferase reporter vector, PRL-TK-pMIR-circCCAR1 or PRL-TK-pMIR-WTAP 3’UTR, miR-127-5p mimics or miR-NC. After 48 hours of incubation, the firefly and Renilla luciferase activities were examined by a Double luciferase reporter gene detection kit (RG027, Beyotime) according to the manufacturer’s instructions.

**Exosome isolation and characterization**

Exosomes were isolated from the plasma of HCC patients by an ExoQuick Plasma Prep with Thrombin kit (SBI, USA), and the culture medium of HCC cell lines was isolated by an ExoQuick TC kit (SBI) according to the manual. Next, transmission electron microscopy (TEM) was used to examine the exosomes. The size distribution was identified by a U30 Flow NanoAnalyzer (NanoFCM, Inc., China). The characterization of the exosomes was also identified by the protein levels of TSG101 (ab133586, Abcam) and CD63 (ab68418, Abcam).

**Exosome incubation with CD8+ T cells**

Exosomes (500 μg) derived from the HCC cell lines were placed into 12-well plates and were cocultured with preactivated CD8+ T cells (1 × 10^5^ per/well). After incubation for 72 hours, flow cytometry, qRT-PCR and Western blotting were used to analyse CD8+ T cells. For exosome tracking, **exosomes** labeled with PKH26 red fluorescent membrane linker dye (Sigma‒Aldrich) were cocultured with activated CD8+ T cells for 12 hours. Fluorescence microscopy was used to observe the uptake process of exosomes in preactivated CD8+ T cells.

**Enzyme-linked Immunosorbent Assay (ELISA)**

After incubation with exosomes for 48 hours, the culture supernatant of preactivated CD8+ T cells was collected. IFN‐γ and TNF-α in the culture supernatant of T cells were measured by ELISA kits (Beyotime) according to the manual. The absorbance at 450 nm was detected by an automatic microplate reader.

**Flow cytometry staining**

CD8 + T cells were incubated with live dead IR-dye (Life Technologies, L34975) at 1:1500 concentration. Cells were washed and surface markers were blocked by incubating cells in PBS supplemented with 10% FBS and Human TruStain FcX™ (Biolegend, 422302) for 15 min. Cells were then stained with Alexa Fluor® 647 Anti-PD1 antibody (Abcam, ab279695), Alexa Fluor® 647 Anti-TIM 3 antibody (ab233059), Alexa Fluor® 647 Anti-LAG-3 antibody (ab225486), Alexa Fluor® 488 Anti-TIGIT antibody (CST, #40129). Cells were fixed with 4% paraformaldehyde for 15 min and then resuspended in PBS for analysis on flow cytometry.

**CD8+ T cell proliferation assays**

The proliferative ability of CD8+ T cells was evaluated by detecting the expression of Ki67 in CD8+ T cells. After fixed with 4% paraformaldehyde for 30 min and permeabilized with 0.1% Triton X-100 for 2 min, the cells were washed and resuspended with PBS buffer. An Alexa Fluor® 488 Anti-Ki67 antibody (ab197234) was added to a CD8+ T cell suspension and incubated in the dark for 30 min. After washing, the expression of Ki67 was detected by flow cytometry.

**TUNEL staining**

The apoptosis level of CD8+ T cells was evaluated by TUNEL staining. After fixed with 4% paraformaldehyde for 30 min and permeabilized with 0.1% Triton X-100 for 2 min, the cells were washed and resuspended with PBS buffer. The CD8+ T cell suspension were then incubated with TUNEL reaction reagent (Beyotime, Beijing, China) for 1h. After washing, the staining cells were detected by flow cytometry.

**Supplementary Figure 1**

**
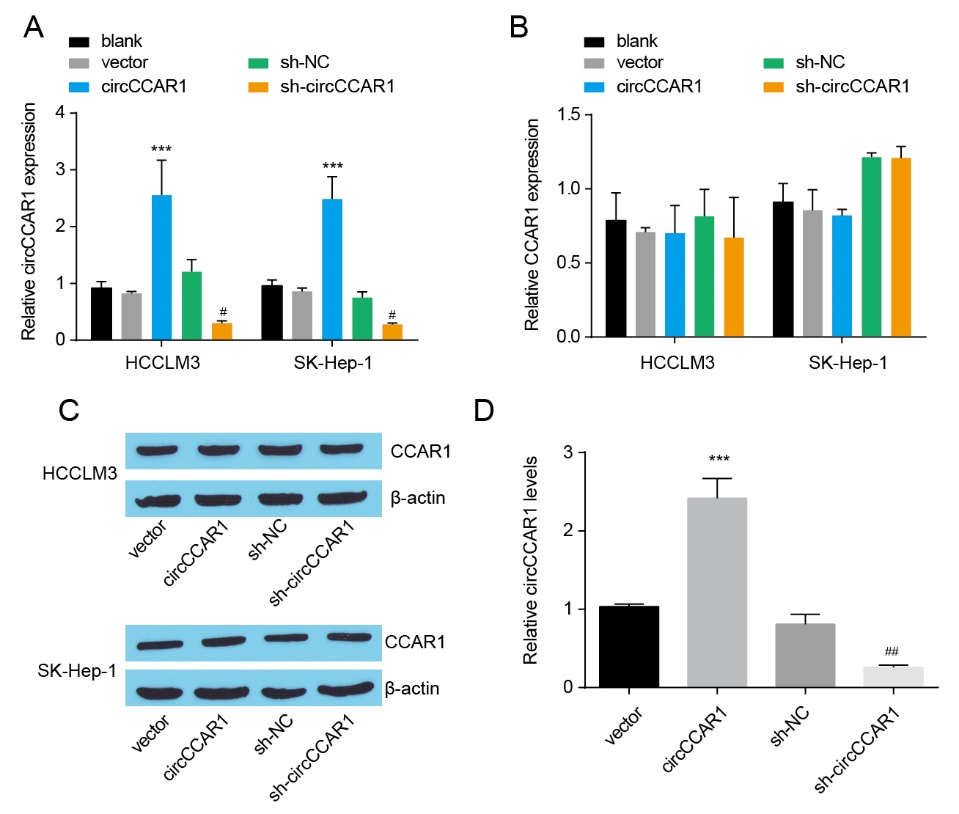
**

**Figure S1 Transfection efficiency validation.** (A) The expression of circCCAR1 in HCCLM3 and SK-Hep-1 cells after circCCAR1 overexpression or depletion. ***p <0.001 vs. vector; #p <0.05 vs. sh-NC. (B-C) The mRNA and protein expression of CCAR1 in HCCLM3 and SK-Hep-1 cells after circCCAR1 overexpression or depletion. (D) Establishment of stable HCCLM3 cells with circCCAR1 overexpression or depletion. ***p <0.001 vs. vector; ##p <0.01 vs. sh-NC.

**Supplementary Figure 2**

**
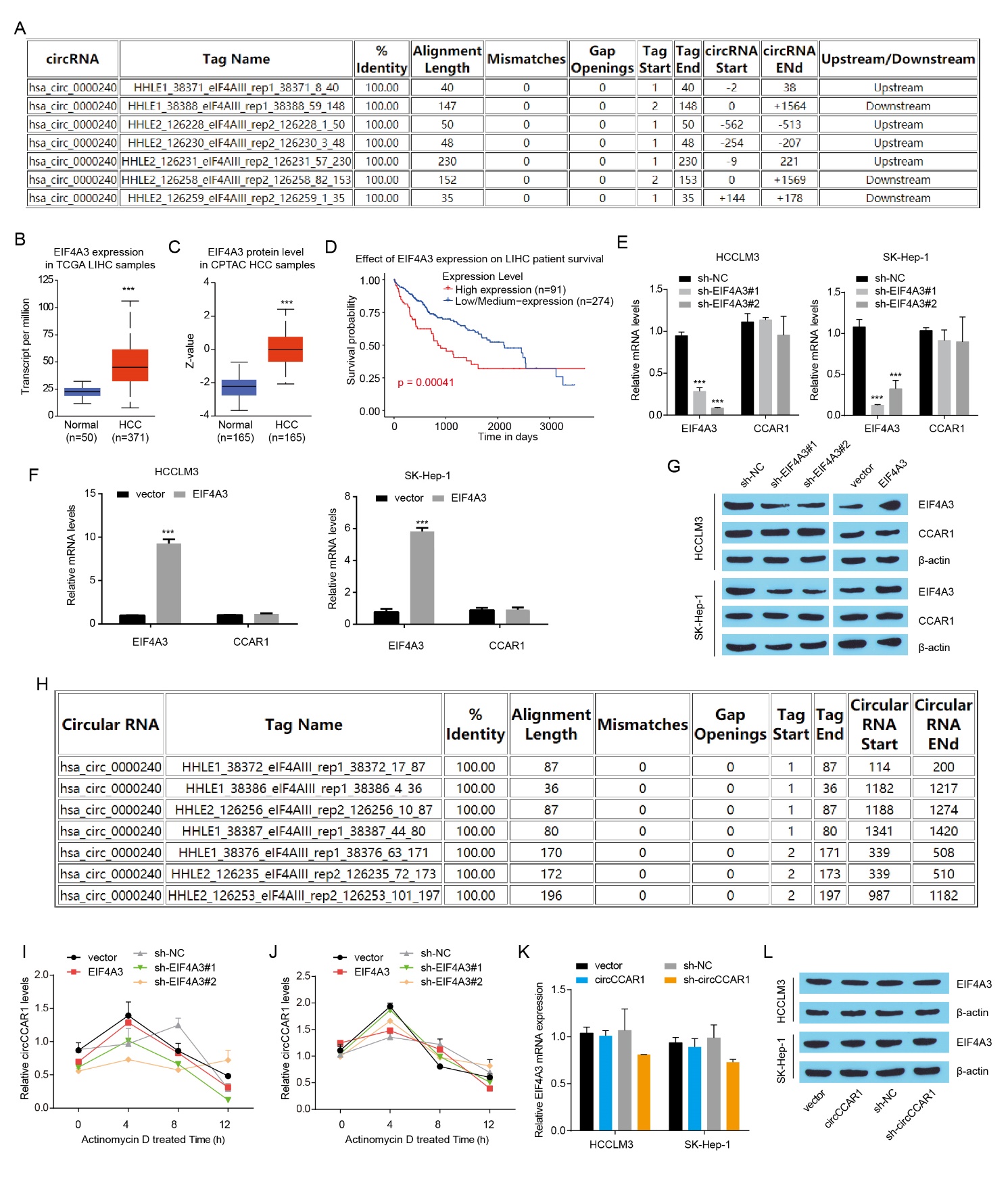
**

**Figure S2 EIF4A3 is implicated in the cyclization and cytoplasmic export of circCCAR1.** (A) Prediction of the putative EIF4A3 binding sites in circCCAR1 pre-mRNA by the CircInteractome database. (B) EIF4A3 expression in TCGA HCC samples and normal samples. ***p <0.001 vs. normal. (C) EIF4A3 protein levels in CPTAC HCC samples and normal samples. ***p <0.001 vs. normal. (D) Kaplan–Meier curve of the correlation between circCCAR1 expression and overall survival (OS) in TCGA HCC patients. (E-F) The mRNA expression of EIF4A3 and CCAR1 in HCCLM3 and SK-Hep-1 cells transfected with EIF4A3 shRNA or EIF4A3 overexpression plasmid. ***p <0.001 vs. sh-NC or vector. (G) The protein expression of EIF4A3 and CCAR1 in HCCLM3 and SK-Hep-1 cells transfected with EIF4A3 shRNA or EIF4A3 overexpression plasmid. (H) Prediction of the putative EIF4A3 binding sites in circCCAR1 by the CircInteractome database. (I-J) Stability of circCCAR1 in EIF4A3-overexpressing or EIF4A3 knockdown HCC cells treated with actinomycin D. (K-L) The mRNA and protein expression of EIF4A3 in HCCLM3 and SK-Hep-1 cells after circCCAR1 overexpression and knockdown.

**Supplementary Figure 3**

**
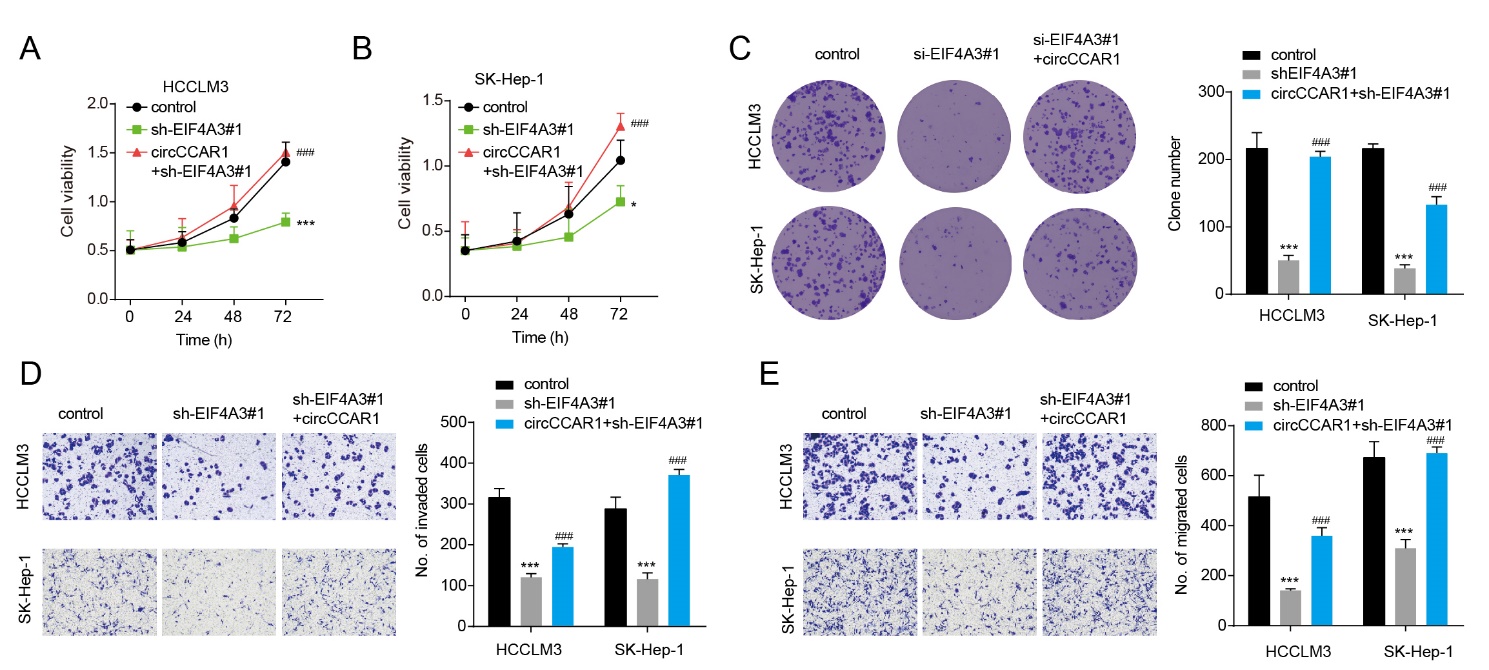
**

**Figure S3 EIF4A3 regulates the growth, migration and invasion of HCC cells by increasing circCCAR1.**

(A-B) A CCK-8 assay was conducted in HCCLM3 and SK-Hep-1 cells after circCCAR1 overexpression or EIF4A3 knockdown. (C) A colony formation assay was conducted in HCCLM3 and SK-Hep-1 cells after circCCAR1 overexpression or EIF4A3 knockdown. (D-E) The migration and invasion abilities of HCC cells after circCCAR1 overexpression or EIF4A3 knockdown were assessed using Transwell assays. *p <0.05, ***p <0.001 vs. control; ###p <0.001 vs. sh-EIF4A3#1.

**Supplementary Figure 4**

**
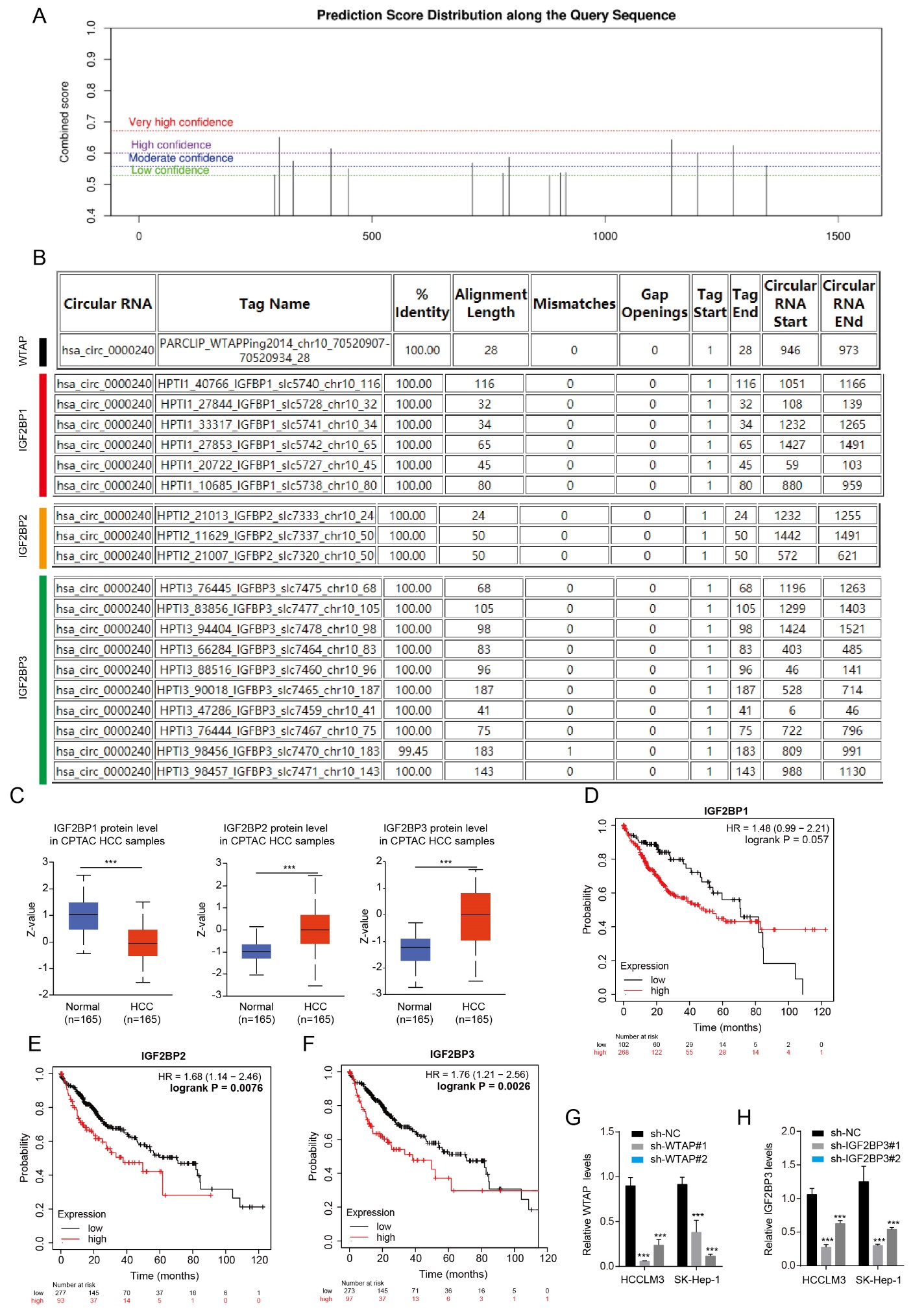
**

**Figure S4 WTAP and IGF2BP3 are related to the stabilization of circCCAR1 in a m6A modification-dependent manner.** (A) The m6A modification site of circCCAR1 predicted by SRAMP website tools. (B) Prediction of the putative WTAP and IGF2BP binding sites in circCCAR1 by the CircInteractome database. (C) IGF2BP1, IGF2BP2, and IGF2BP3 protein levels in CPTAC HCC samples and normal samples. ***p <0.001. (D-F) The association of IGF2BP1, IGF2BP2, and IGF2BP3 expression levels and overall survival in HCC patients. (G) The relative expression of WTAP in HCC cells after WTAP knockdown. (H) The relative expression of IGF2BP3 in HCC cells after IGF2BP3 knockdown. ***p <0.001 vs. sh-NC.

**Supplementary Figure 5**

**
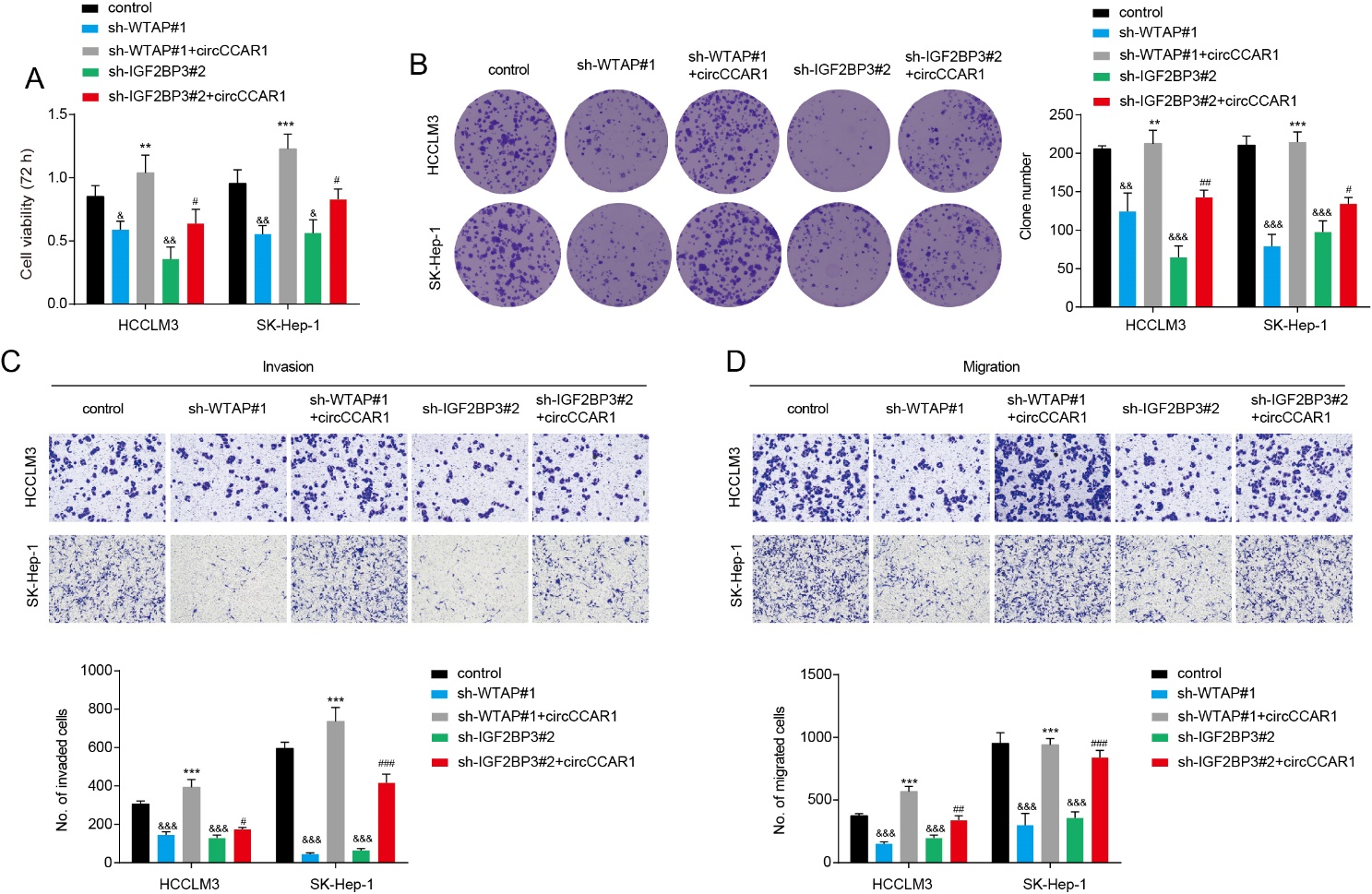
**

**Figure S5 The WTAP-IGF2BP3 axis regulates the growth, migration and invasion of HCC cells by stabilizing circCCAR1.** (A) A CCK-8 assay was conducted in HCCLM3 and SK-Hep-1 cells after WTAP knockdown, IGF2BP3 knockdown, or circCCAR1 overexpression. (B) A colony formation assay was conducted in HCCLM3 and SK-Hep-1 cells. (C-D) The migration and invasion abilities of HCC cells were assessed using Transwell assays. &p <0.05, &&p <0.01, &&&p <0.001 vs. control; **p <0.01, ***p <0.001 vs. sh-WTAP; #p <0.05, ##p <0.01, ###p <0.001 vs. sh-IGF2BP3#2.

**Supplementary Figure 6**


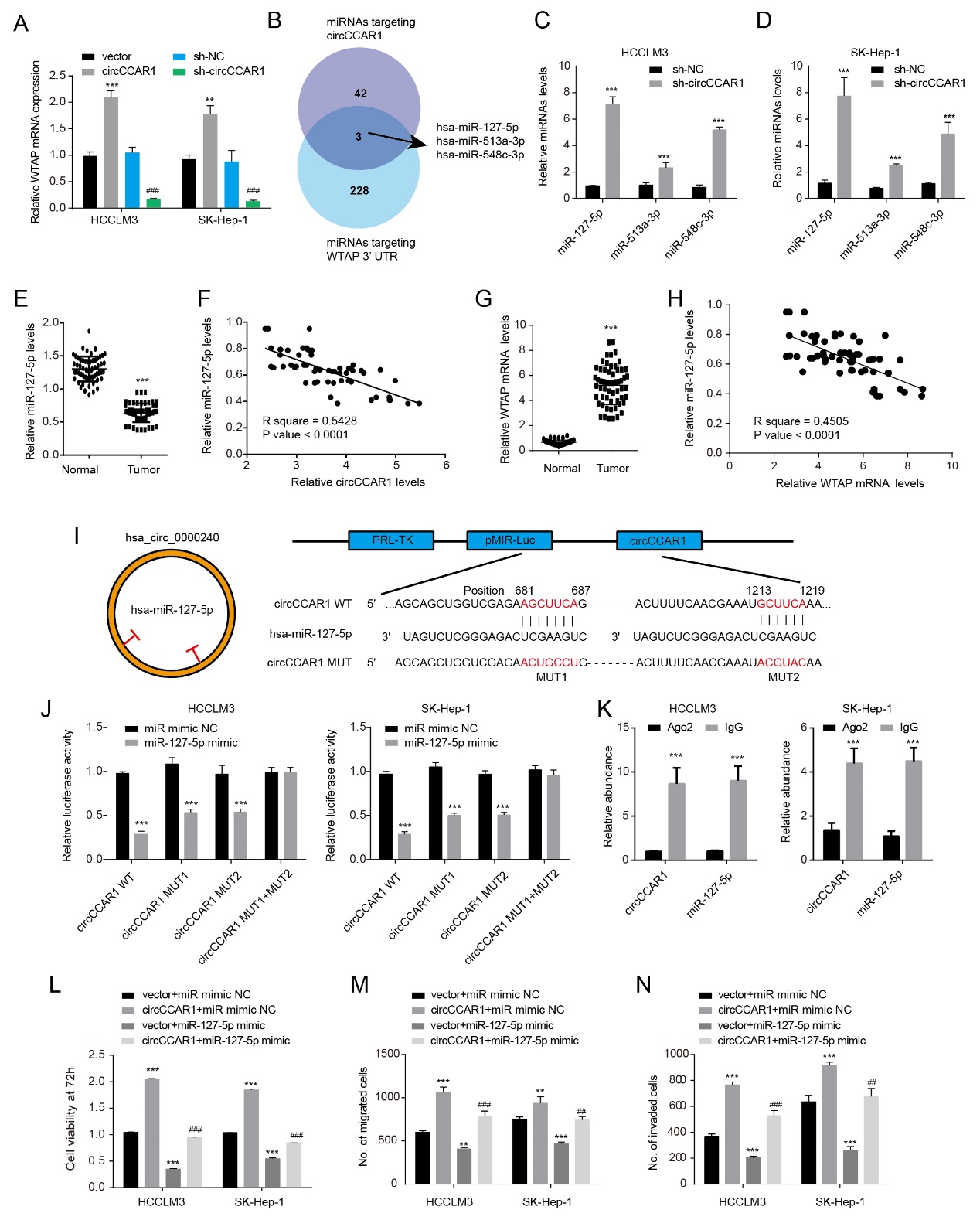


**Figure S6 CircCCAR1 acted as a sponge for miR-127-5p in HCC cells.** (A) WTAP mRNA level in HCC cells with circCCAR1 overexpression of knockdown. ***p <0.001 vs. vector; ###p <0.001 vs. sh-NC. (B) Venn diagram showed the intersection of TargetScan and CircInteractome in potential miRNA binding sites of circCCAR1. (C-D) MiR-127-5p, miR-513a-3p and miR-548c-3p expression in HCC cells with circCCAR1 knockdown. ***p <0.001 vs. sh-NC. (E) MiR-127-5p levels in 58 HCC patient tumor tissues and adjacent normal tissues. ***p <0.001 vs. Normal. (F) A negative correlation between miR-127-5p levels and circCCAR1 expression was found in 58 HCC patient tumor tissues. (G) WTAP levels in 58 HCC patient tumor tissues and adjacent normal tissues. ***p <0.001 vs. Normal. (H) A negative correlation between miR-127-5p levels and WTAP expression was found in 58 HCC patient tumor tissues. (I) The two potential binding sites of miR-127-5p in circCCAR1 was displayed. (J) Comparison of the luciferase activity of circCCAR1 after treatment with miR-127-5p mimics in HCCLM3 and SK-hep-1 cells. ***p <0.001 vs. miR mimic NC. (K) RIP analysis of the enrichment levels of circCCAR1 and miR-127-5p pulled down from Ago2 or IgG protein in HCCLM3 and SK-hep-1 cells. ***p <0.001 vs. IgG. (L) The cell viability of HCC cells was detected after co-transfection with circCCAR1 or miR-127-5p mimic by CCK8 assay. (M-N) The migration and invasion abilities of HCC cells co-transfected with circCCAR1 or miR-127-5p mimic were measured by Transwell migration and invasion assays. **p <0.01, ***p <0.001 vs. vector+miR mimic NC; ##p <0.01, ###p <0.001 vs. circCCAR1+miR mimic NC.

**Supplementary Figure 7**


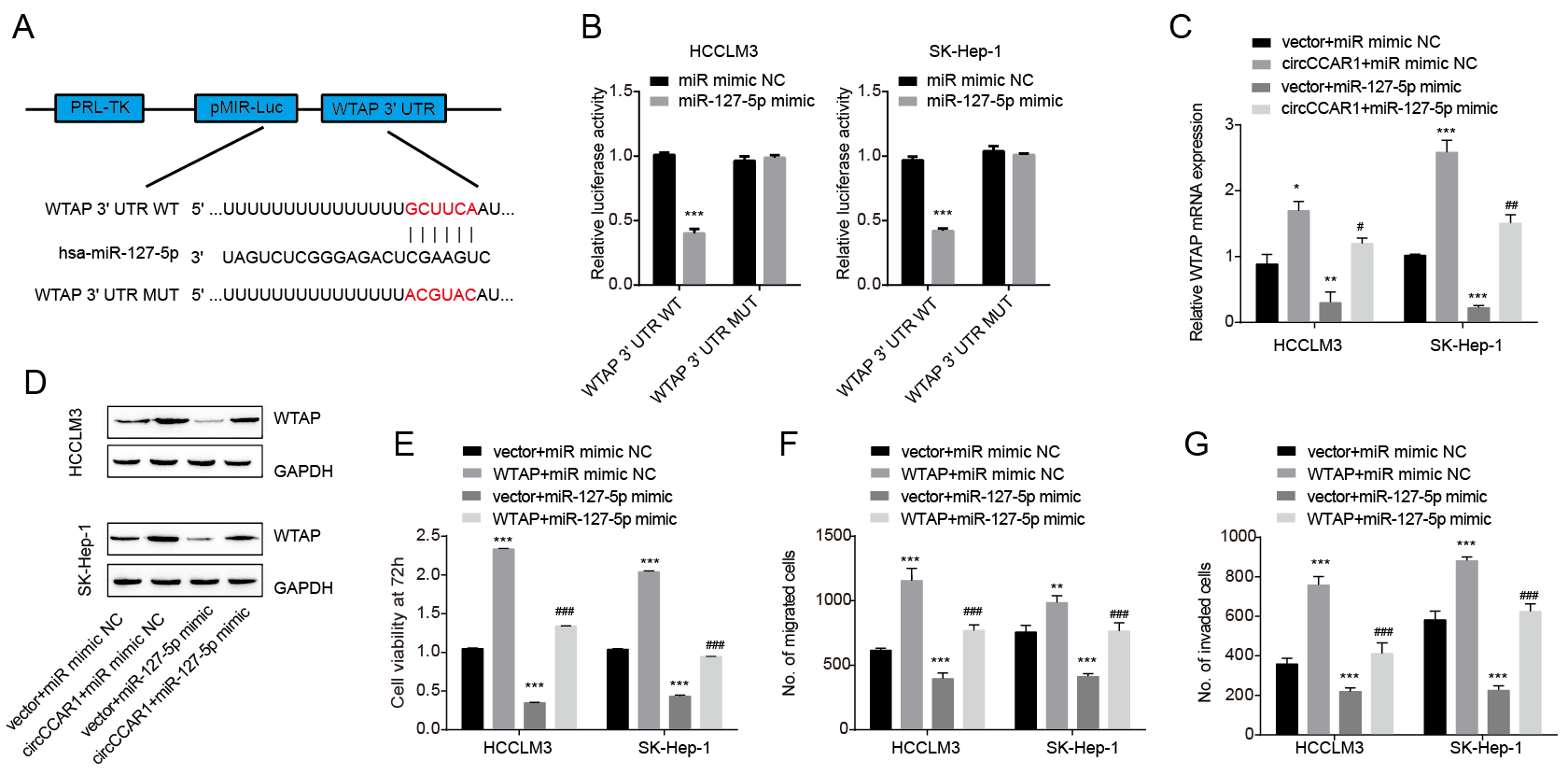


**Figure S7 WTAP is a direct target of miR-127-5p in HCC.** (A) Schematic illustration of WTAP 3’UTR WT and MUT were displayed. (B) Comparison of the luciferase activity of circCCAR1 after treatment with miR-127-5p mimics in HCCLM3 and SK-hep-1 cells. ***p <0.001 vs. miR mimic NC. (C-D) WTAP mRNA and protein level in HCC cells with circCCAR1 overexpression or miR-127-5p overexpression. (E) The cell viability of HCC cells was detected after co-transfection with circCCAR1 or miR-127-5p mimic were measured by CCK8 assay. (F-G) The migration and invasion abilities of HCC cells co-transfected with circCCAR1 or miR-127-5p mimic were measured by Transwell migration and invasion assays. *p <0.05, **p <0.01, ***p <0.001 vs. vector+miR mimic NC; ##p <0.01, ###p <0.001 vs. vector+miR-127-5p mimic.

**Supplementary Figure 8**


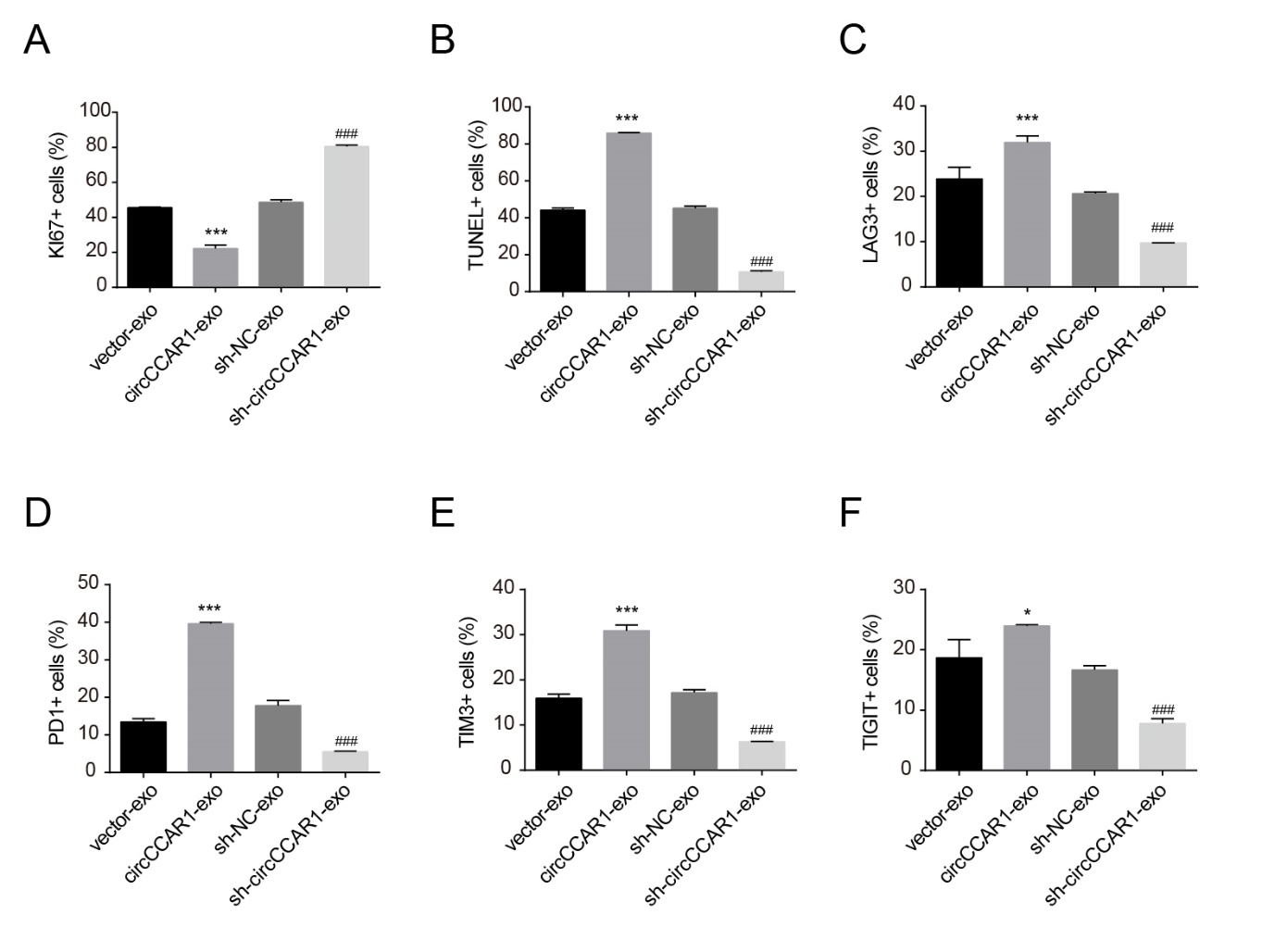


**Figure S8 Exosomal circCCAR1 inhibited the proliferation, promoted the apoptosis, and increased LAG3, PD1, TIM3, and TIGIT expression in cell surface of CD8+ T cells.** Activated CD8+ T cells were co-cultured with indicated exosomes for 72 hours. (A) The proliferation ability of CD8+ T cells was evaluated by measuring the expression of Ki67 in CD8+ T cells by flow cytometry. (B) TUNEL assay was used to evaluate the apoptosis of CD8+ T cells. (C-D) The surface expression of LAG3, PD1, TIM3, and TIGIT in CD8+ T cells was measured by flow cytometry. *p <0.05, ***p <0.001 vs. vector-exo; ###p <0.001 vs. sh-NC-exo.

**Supplementary Figure 9**

**
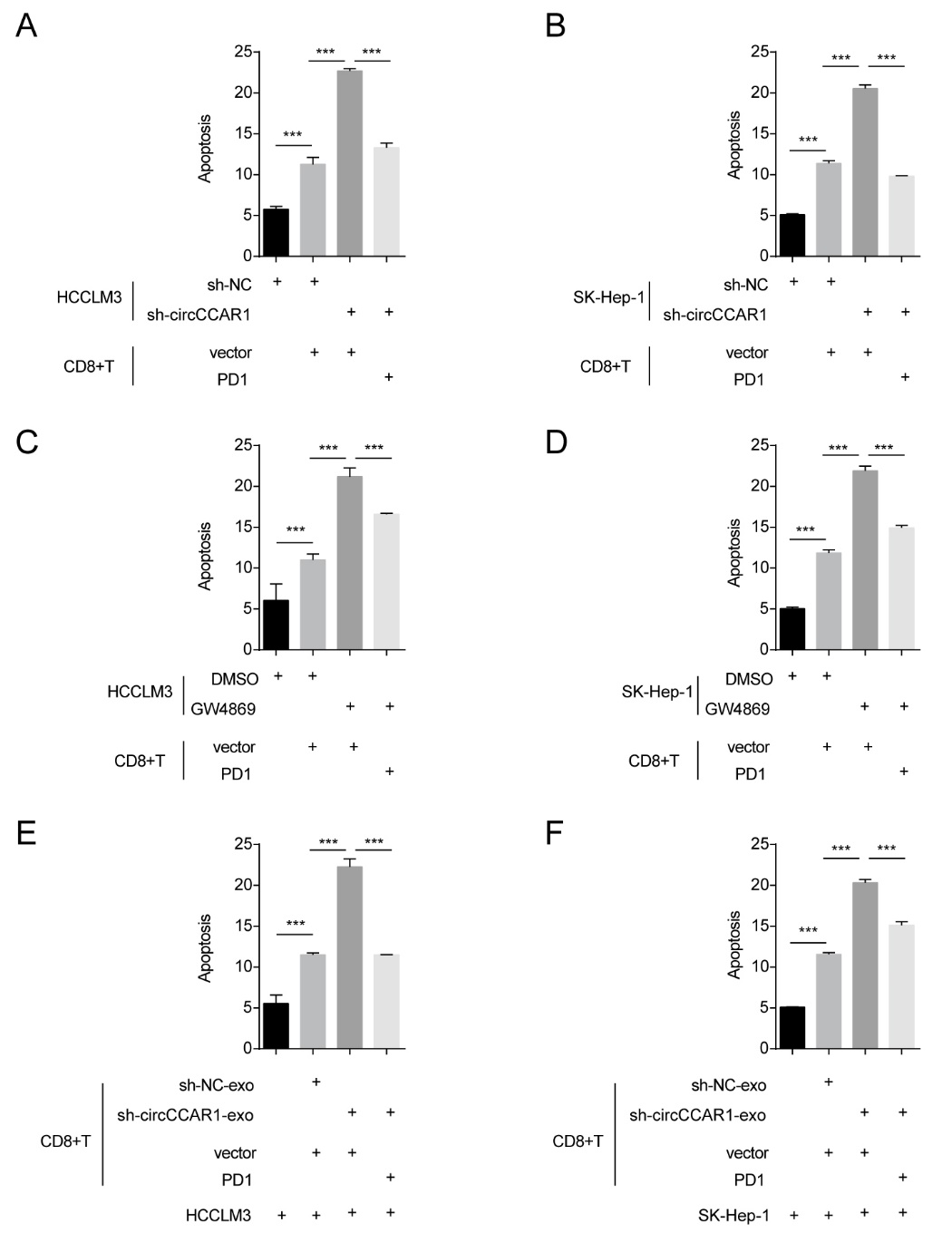
**

**Figure S9 Exosomal circCCAR1 inhibited the apoptosis of HCC cells induced by CD8^+^ T cells by targeting PD1.** (A-B) HCC cells expressing control shRNA or circCCAR1 shRNA were cocultured with activated CD8^+^ T cells with or without PD-1 overexpression for 48 h. The percentage of apoptotic HCC cells was determined using flow cytometry analysis. (C-D) HCC cells treated with GW4869 were cocultured with activated CD8^+^ T cells with or without PD-1 overexpression for 48 h. The percentage of apoptotic HCC cells was examined using flow cytometry analysis. (E-F) HCC cells were cocultured with activated CD8^+^ T cells with exosome treatment or PD-1 overexpression for 48 h. The percentage of apoptotic HCC cells was examined using flow cytometry analysis. ***p <0.001.

**Supplementary Figure 10**

**
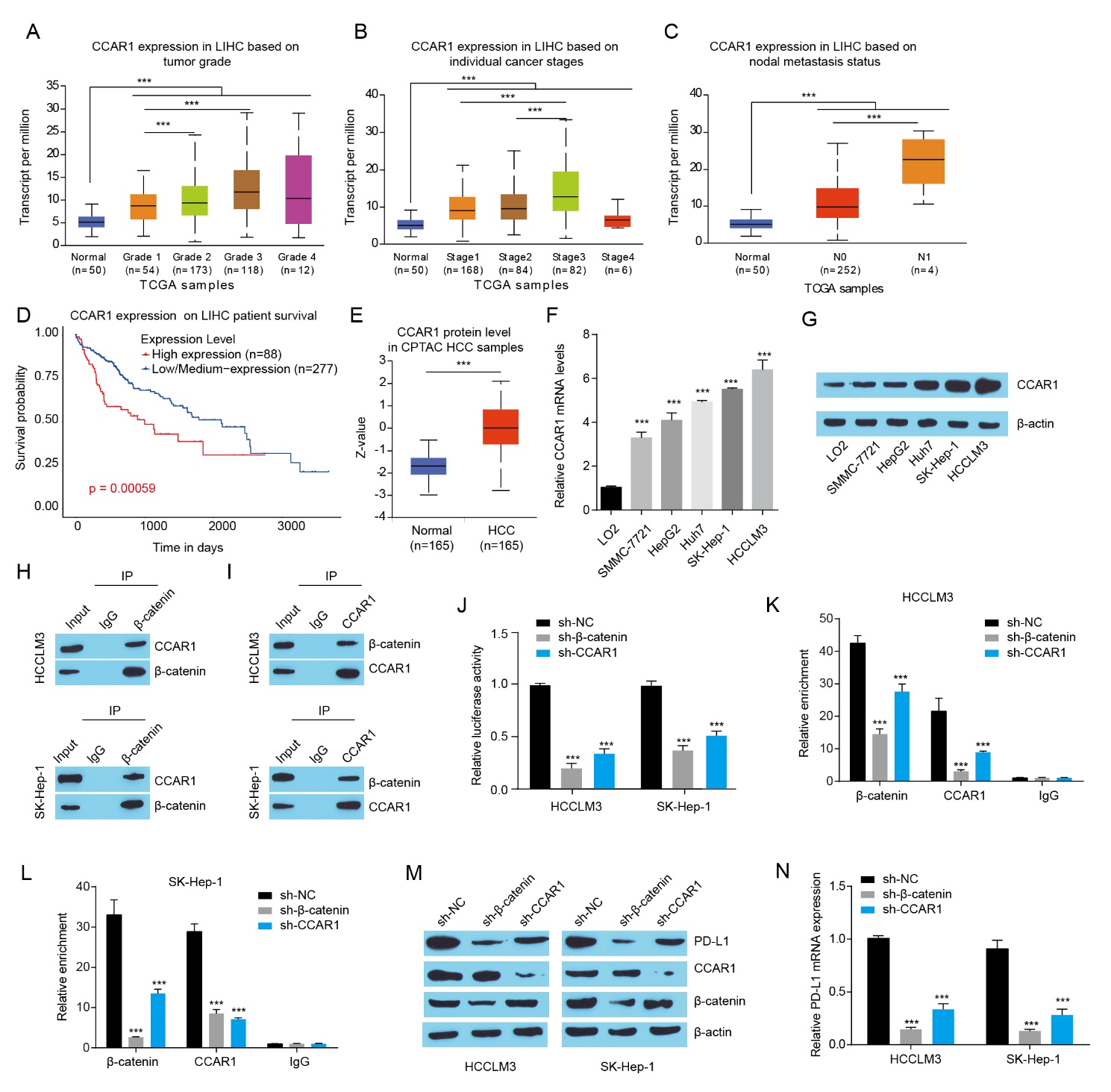
**

**Figure S10 CCAR1** **enhanced PD-L1 expression by interacting with β-catenin.** (A) The expression of CCAR1 mRNA in normal tissue or HCC samples corresponding to tumor grades 1, 2, 3, and 4. ***p <0.001. (B) The expression of CCAR1 mRNA in normal tissue or HCC samples corresponding to individual cancer stages 1, 2, 3, and 4. ***p <0.001. (C) The expression of CCAR1 mRNA in normal tissue or HCC samples corresponding to nodal metastasis status N0 and N1. ***p <0.001. (D) The association of the CCAR1 expression level and overall survival in TCGA HCC patients. (E) The expression of CCAR1 protein in normal tissue of HCC samples. ***p <0.001. (F-G) The expression of CCAR1 mRNA and protein levels in LO2 and HCC cells. ***p <0.001 vs. LO2. (H-I) Coimmunoprecipitation (IP) was performed on HCCLM3 or SK-Hep-1-cell extracts using the indicated antibodies against β-catenin or CCAR1 or normal IgG. (J) Luciferase activity in HCCLM3 and SK-Hep-1 cells cotransfected with a luciferase reporter and siRNA for CCAR1 or β-catenin. (K-L) HCCLM3 and SK-Hep-1 cells with the indicated transfection were subjected to ChIP assays with CCAR1 and β-catenin antibodies. (M) The protein levels of β-catenin, CCAR1 and PD-L1 in HCCLM3 and SK-Hep-1 cells after β-catenin or CCAR1 knockdown. (N) The expression of CCAR1 mRNA in HCC cells after CCAR1 or β-catenin depletion. ***p <0.001 vs.sh-NC.

**Supplementary Table S1.** Correlation between circCCAR1 expression and clinicopathologic parameters of patients with HCC (n = 58)

| **Variables** | circCCAR1 **High (n=29)** | circCCAR1 **Low (n=29)** | **p value** |
| --- | --- | --- | --- |
| Gender |  |  | 0.79 |
| Male | 13 | 11 |  |
| Female | 16 | 18 |  |
| Age (year) |  |  | 0.60 |
| <60 | 11 | 14 |  |
| ≥60 | 18 | 15 |  |
| Tumor size (cm) |  |  | 0.03 |
| <5 | 9 | 18 |  |
| ≥5 | 20 | 11 |  |
| Tumor grade |  |  | <0.01 |
| I | 7 | 20 |  |
| II, III | 22 | 9 |  |
| TNM stage |  |  | 0.02 |
| I/II | 11 | 16 |  |
| III/IV | 18 | 13 |  |
| Vascular invasion |  |  | <0.01 |
| No | 10 | 21 |  |
| Yes | 19 | 8 |  |

**Supplementary Table S2:** Data of sequences for PCR and shRNA in this study

| **Gene** | **Sequences (5’-3’)** |
| --- | --- |
| GAPDH | Forward: ACAACTTTGGTATCGTGGAAGG |
|  | Reverse: GCCATCACGCCACAGTTTC |
| CircCCAR1 | Forward: TGAGGATGAAGAAGATGTCCCA |
|  | Reverse: TGCAGTAATTTCCCAGCTGA |
| CCAR1 | Forward: CTGATGGCTAGCCCTAGTATGGA |
|  | Reverse: TGCCTTTCATGCCCACTAAAA |
| U1 | Forward: CAGGGCGAGGCTTATCCA |
|  | Reverse: GGAAATCGCAGGGGTCAGCACATCC |
| PD-L1 | Forward: GCCGAAGTCATCTGGACAAG |
|  | Reverse: TCTCAGTGTGCTGGTCACAT |
| EIF4A3 | Forward: CCCTCACCACAATGACAGCA |
|  | Reverse: TGACCCACGCAGGTTAAACA |
| EP300 | Forward: AGCCAACGCGGCCTAAACT |
|  | Reverse: TCACCACCATTGGTTAGTCCC |
| WTAP | Forward: ACTGGCCTAAGAGAGTCTGAAG |
|  | Reverse: GTTGCTAGTCGCATTACAAGGA |
| IGF2BP3 | Forward: CCATAGAAGTTGAGCACTCGGTCC |
|  | Reverse: TCTCCACCACTCCATACTGGACTAG |
| hnRNPA2B1 | Forward: CAGGGTAGTTGAGCCAAAACG |
|  | Reverse: TTCCAGACTGCCTATCGGTAA |
| PD1 | Forward: ATGCAGATCCCACAGGCGCC |
|  | Reverse: TCAGAGGGGCCAAGAGCAGTG |
| CCAR1 promoter | Forward: CAGAACGTCAGCCAGTAAGC |
|  | Reverse: GCTACCCAAAAGATCGCTCC |
| Divergent primers for circCCAR1 | Forward: AAACGGAGAAAATCAGGCGA |
|  | Reverse: GGCGCCTTAGTTCCATCATG |
| Convergent primers for circCCAR1 | Forward: GACATGATGGAACTAAGGC |
|  | Reverse: GCACTGTATAAGTGGTCAGC |
| miR-127-5p | Forward: GCCGAGCTGAAGCTCAGAGG |
|  | Reverse: CTCAACTGGTGTCGTGGA |
| U6 | Forward: CTCGCTTCGGCAGCACATATACT |
|  | Reverse: ACGCTTCACGAATTTGCGTGTC |
| miR-513a-3p | Forward: TAAATTTCACCTTTCTGAG AAGG |
|  | Reverse: GCGAGCACAGAATTAATACGAC |
| miR-548c-3p | Forward: ACACTCCAGCTGGGCAAAAATCTCAAT |
|  | Reverse: CTCAACTGGTGTCGTGGA |
| sh-NC | GCTCTACTTCGACGACAAGAT |
| sh-circCCAR1 | CTACTTCTTCTACAGGGTCAACACT |
| sh-EP300#1 | CGGAAACAGTGGCACGAAGAT |
| sh-EP300#2 | GCGGAATACTACCACCTTCTA |
| sh-EIF4A3#1 | GGAAGACATGACTAAAGTGGA |
| sh-EIF4A3#2 | GCAATCCAGCAACGAGCAATT |
| sh-WTAP#1 | AAGGTTCGATTGAGTGAAACA |
| sh-WTAP#2 | GAAGCATATGTACAAGCTT |
| sh-IGF2BP3#1 | CGGTGAATGAACTTCAGAATT |
| sh-IGF2BP3#2 | GGTGAAACTTGAAGCTCATAT |
| sh-hnRNPA2B1#1 | GCAACAAGGAAGGGCATCTTGGTTA |
| sh-hnRNPA2B1#2 | CAAGGAAGGGCATCTTGGTTATGAA |
| miR-127-5p mimic | UAGUCUCGGG AGACUCGAAGUC |
| miR-NC | UAGUCUCGGGAGAC UCACUACC |
